# Craniodental divergence associated with bite force between hybridizing pine squirrels (*Tamiasciurus*)

**DOI:** 10.1371/journal.pone.0284094

**Published:** 2023-04-06

**Authors:** Dylan M. Poorboy, Jonathan J.-M. Calède, Andreas S. Chavez

**Affiliations:** 1 Department of Evolution, Ecology and Organismal Biology, The Ohio State University, Columbus, OH, United States of America; 2 The Ohio State University at Marion, Marion, OH, United States of America; 3 Translational Data Analytics Institute, The Ohio State University, Columbus, OH, United States of America; Ecole Normale Supérieure de Lyon, FRANCE

## Abstract

Bite force can be a limiting factor in foraging and can significantly affect the competitive ability and lifetime fitness of mammals. *Tamiasciurus* squirrels feed primarily on conifer seeds and have a strong bite force to mechanically extract seeds from conifer cones with their mouths. In the North Cascades region, Douglas squirrels (*Tamiasciurus douglasii*) and red squirrels (*T*. *hudsonicus*) occupy ecologically different forests with different hardnesses in conifer cones. The ranges of these species overlap in a narrow hybrid zone where these forests meet near the crest of the North Cascades. We examined interspecific divergence in dietary ecomorphology in allopatry, in sympatry within the hybrid zone, and between hybrids and each parental species. We focused on three craniodental traits, including the incisor-strength index as a proxy measure for maximal bite force, cranial-suture complexity, and mandible shape. We find that these sister squirrel species differ in bite force and suture complexity in allopatry and sympatry and that mandible shape changes with the expected hardness of accessed food items, but is not significantly different between species. Furthermore, we find that hybrids display morphologies that overlap with hybrid zone red squirrels but not with hybrid zone Douglas squirrels. This work shows how important ecological processes at shallow evolutionary timescales can impact the divergence of morphological traits in taxa with extreme conservation of craniomandibular shape.

## Introduction

The ecological processes that generate morphological divergence can be challenging to discern in comparative studies focused on species across deep taxonomic timescales. Ecomorphology can be the product of several, at times opposing, selective processes that may not persist through the entire evolutionary history of deep lineages. At shallow timescales, different processes, including divergent ecological selection of morphology and behavior, hybridization, and interspecific competition, can be important initiators of ecomorphological diversity [1–3]. Hybrid zones representing secondary contact and hybridization between ecologically divergent species are practical arenas to investigate how these three different processes impact variation on ecomorphology at shallow timescales. In hybrid zones that span ecological transitions, differences in phenotypic variation between species is subject to the homogenizing effects of gene flow and its counterposing force, divergent selection [4–6]. Furthermore, interspecific competition within hybrid zones can potentially drive the divergent evolution through character displacement [7–9] or, in contrast, phenotypic convergence driven by shared ecological pressures [10–12].

Hybridization, in the absence of evolutionary forces, can have significant consequences on phenotypic variation. Hybridization leads to novel epistatic interactions between differentiated genomes, which may allow for a change in morphospace occupation due to a relaxation of trait covariation [13, 14]. Thus, hybridization can immediately lead to (1) hybrid phenotypes that are intermediate between the parental phenotypes [15, 16]; (2) hybrid phenotypes that resemble one of the parental phenotypes, reflecting genetic dominance [17–19]; or (3) transgressive hybrid phenotypes that are more extreme than either parental phenotype [20–24]. The consequences of evolutionary forces on hybrid phenotypes are also varied and range from strong selection against hybrid morphologies [25] to selection that favors novel hybrid morphologies [2, 3].

Ecomorphological traits associated with foraging are routinely under strong natural selection for functional performance and illustrate the combination of both precise local adaptation and extreme evolutionary diversification [26–29]. In mammals, several craniodental traits are associated with generating and sustaining maximal bite force. First, the incisor strength index is a reliable proxy for maximal bite force in rodents [30]. This index is based on a rectangular cross-section of the incisor and its ability to resist a bending force, which allows it to be a valuable estimator of bite force where *in vivo* measurements are challenging to acquire or consistently replicate due to variable behavior [30]. This index was developed from interspecific studies in deeper evolutionary time than is addressed herein; however, we expect it to be functional as a proxy measure of comparing relative biting strength, although it may not be directly translatable to units of force. Next, the suture lines of the cranium reveal the counterforce stresses that the cranium receives from biting on food items [31–35]. Consequently, suture complexity is positively associated with a greater ability to absorb and redistribute the shocks that result from stronger bite force [36, 37]. Finally, mandible shape is another demonstrated predictor of bite force [38] and may influence masticatory muscle attachment and mechanical levers, with the expectation that more durophagous animals will have larger or more robust mandibular processes and higher mechanical lever advantages at the incisors [28, 39–41].

**Fig 1:**
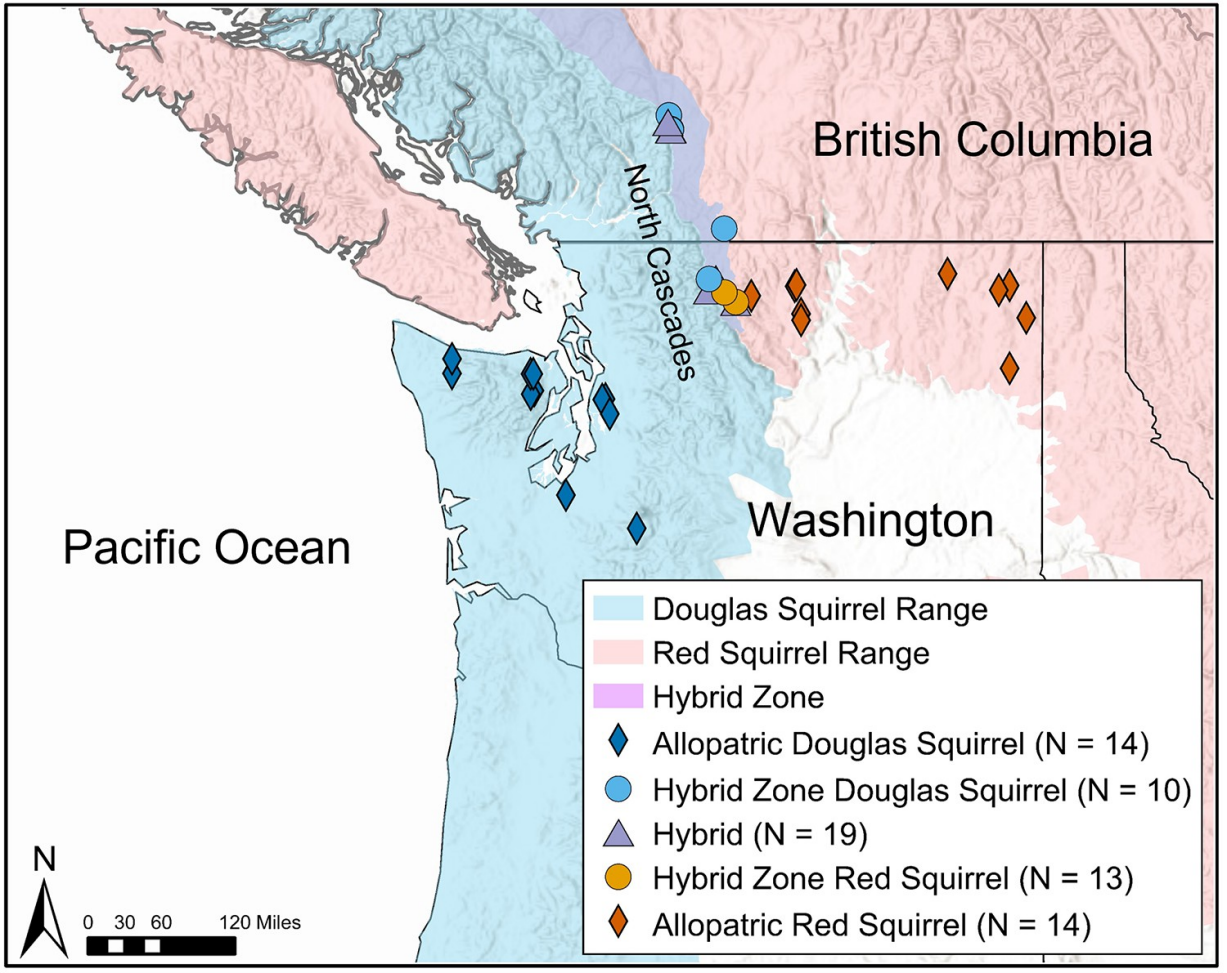
Map indicating approximate range boundaries for Douglas squirrels (blue), red squirrels (red), and the hybrid zone (purple) in the North Cascades region. Range shapefiles developed from published descriptions [49, 50] by A.S. Chavez. Symbols indicate sampling localities. Some specimens within the hybrid zone geographically overlap, see S1 Table for complete specimen details. Basemap reprinted from Terrain With Labels under a CC BY license, with permission from ESRI, original copyright 2016.

The hybrid zone between ecologically divergent Douglas squirrels (*Tamiasciurus douglasii*) and red squirrels (*T*. *hudsonicus*) represents an exemplary opportunity to study the consequences of ecologically divergent selection, interspecific competition, and hybridization on craniodental traits associated with bite force. This parapatric pair of squirrel species diverged less than a million years ago and are hybridizing in a narrow secondary contact zone near the crest of the North Cascades region in northern Washington and southern British Columbia (Fig 1; [42–44]). *Tamiasciurus* squirrels are strongly associated with coniferous forests, feed primarily on conifer seeds by mechanically removing cone scales with their mouth, are mostly asocial, and will vigorously defend territories to protect their centralized cache of conifer cones [42, 45].

In the greater region outside of the hybrid zone, major habitat differences between Douglas squirrels and red squirrels implicate ecological divergent selection as an important driver of divergence in craniodental traits associated with bite force. Douglas squirrels occupy wet maritime forests on the west side of the Cascade Range, and red squirrels occupy dry continental forests on the east side. These habitat differences correspond with differences in the hardness of conifer cones. In the dry continental forests on the east side, lodgepole pine seeds are an important food for red squirrels and are encased in hard, serotinous (closed) cones. In contrast, all conifer species in the wet maritime forests in the range of Douglas squirrels produce qualitatively softer and more opened cones [46–48]. One of the most striking craniodental differences between these squirrel species that may be associated with their different biomechanical challenges of procuring conifer seeds is the prominent sagittal crest found in red squirrels and the shorter and medio-laterally expanded sagittal “plateau” found in Douglas squirrels [43, 45]. This difference in sagittal crest development is expected to drive a difference in produced bite force for these species, as it relates to muscle attachment space for the temporalis muscle involved in the biting motion. Comparisons of other craniodental traits associated with bite force between these squirrel species living in different forests will help inform whether or not divergent selection has generated greater divergence in other parts of the skull than previously understood.

Within the hybrid zone, observational studies have shown that both parental squirrel species and their hybrids are syntopic and select similar food items in subalpine forests dominated by Engelmann spruce and subalpine fir [45, 51], which are expected to be qualitatively soft cones. Hybrids can also backcross successfully with both parental species [43]. The territorial behavior and overlapping niche of both parental species and their hybrids within the hybrid zone indicate that interspecific competition among all squirrel types is potentially strong, which may have important consequences on access to food resources and, ultimately, the evolution of craniodental morphologies associated with foraging. One potential outcome of intense interspecific competition is the evolution of ecological character displacement in response to selection that lessens resource competition between interacting species and leads to specialization towards different resources [7]. However, character displacement and competitive exclusion in hybrid-zone systems may be prevented by the co-existence and viability of hybrids with intermediate phenotypes [52]. Alternatively, these species may converge in their ecomorphologies due to the selective pressures of a shared food resource. Comparisons of ecomorphologies between hybrid zone populations and allopatric populations for each parental squirrel species and their hybrids can begin to uncover whether interspecific competition or convergent evolution are important factors driving morphological change. Furthermore, comparisons of ecomorphologies between hybrids and both parental species can help reveal how the creative effects of hybridization may lead to ecomorphologies outside of or within the phenotypic range of the parental species.

Here, we conduct a comparative study of bite force strength, suture complexity, and mandible shape to determine whether divergence in these functional traits is consistent with ecological divergent selection, interspecific competition, and hybridization. Specifically, we investigate the following three hypotheses on the patterns of ecomorphological differences associated with bite force: (1) differences between allopatric Douglas squirrels and red squirrels are consistent with divergent selection due to major differences in cone hardness; (2) ecomorphologies between Douglas squirrels and red squirrels in sympatry within the hybrid zone are convergent, consistent with a shared ecology; and (3) hybrid morphologies are overlapping with or intermediate to either parental species due to genetic admixture between the parental species and a shared ecology.

## Results

### Bite force

**Fig 2:**
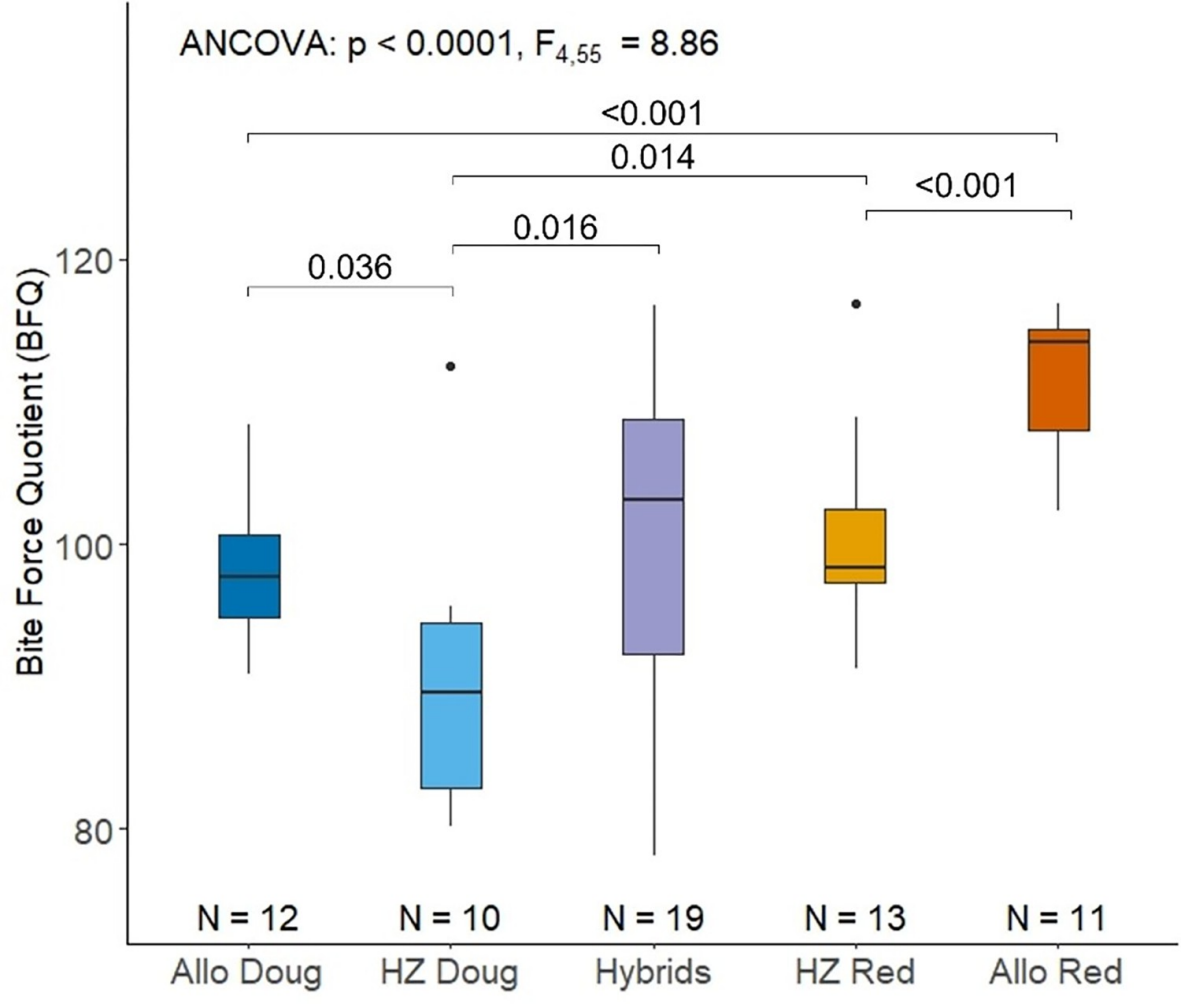
Boxplot showing variation in bite force quotient (BFQ) for each group of squirrels. Values on comparison bars represent significant p-values from pairwise comparisons. Outliers are indicated by black dots. Abbreviations: Allopatric Douglas squirrel, Allo Doug; Hybrid Zone Douglas squirrel, HZ Doug; Hybrid Zone red squirrel, HZ Red; and allopatric red squirrel, Allo Red.

The bite force quotient (BFQ) of the lower incisor differs significantly across squirrel groups (Fig 2; p<0.0001, F_4,55_ = 8.86). Pairwise comparisons reveal that the BFQ is lower for Douglas squirrels than for red squirrels, both in allopatry (p < 0.0001) and in sympatry within the hybrid zone (p = 0.014). The hybrid zone red squirrel population has a lower BFQ than the allopatric red squirrel population (p = 0.014). Similarly, the hybrid zone Douglas squirrel population has a lower BFQ than the allopatric Douglas squirrel population (p = 0.036). The BFQ of hybrids is not significantly different from that of sympatric hybrid-zone red squirrels (p = 0.94), but it is significantly greater than that of sympatric hybrid-zone Douglas squirrels (p = 0.016).

### Suture morphology

**Fig 3:**
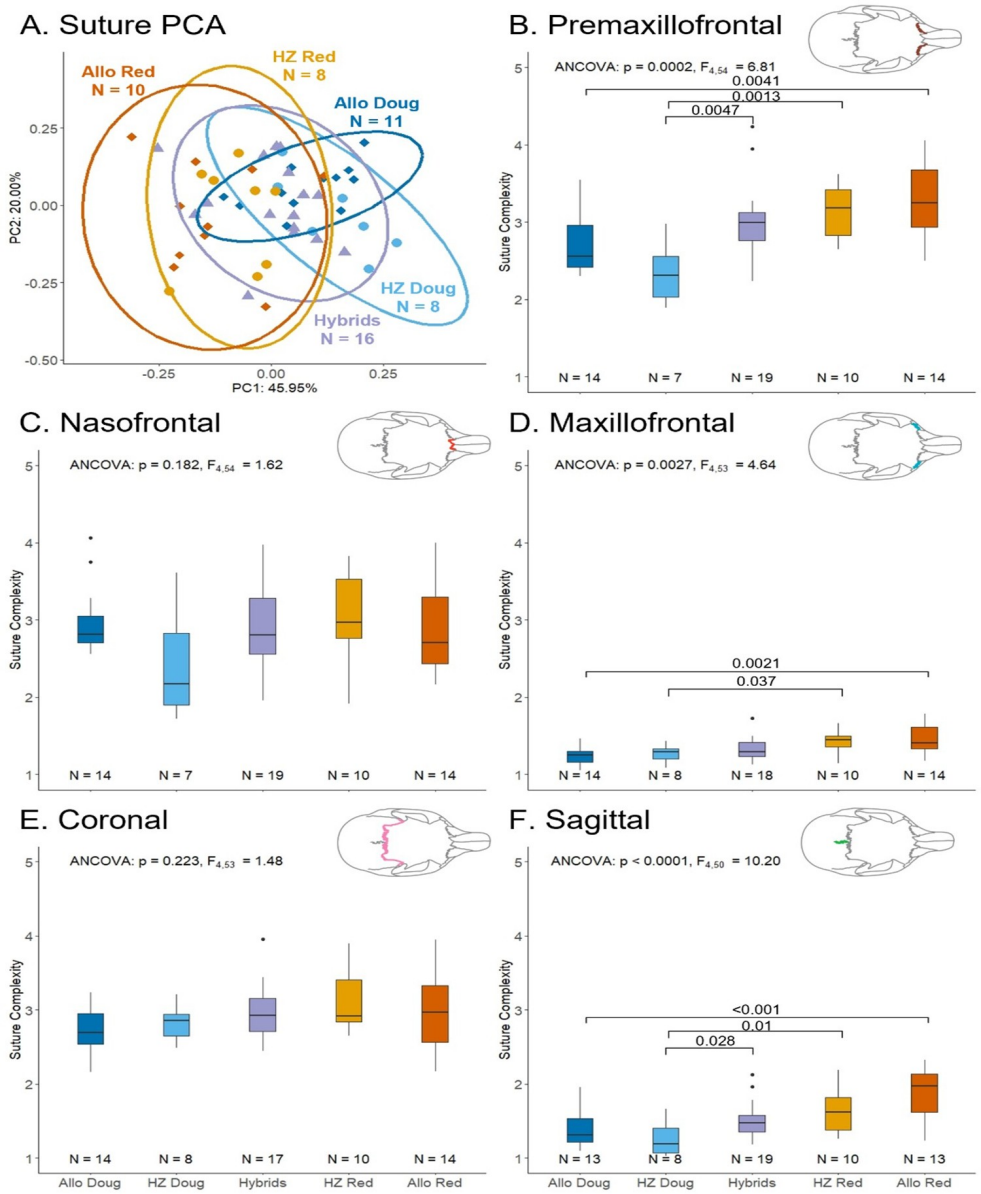
Suture complexity comparisons between *Tamiasciurus* groups. Values on comparison bars represent significant p-values from pairwise comparisons. Outliers are indicated by black dots. Abbreviations are provided in Fig 2. (A) Principal component analysis for all five sutures. (B) ANCOVA for the premaxillofrontal suture. (C) ANCOVA for the nasofrontal suture. (D) ANCOVA for the maxillofrontal suture. (E) ANCOVA for the coronal suture. (F) ANCOVA for the sagittal suture.

We find significant differences in suture complexity (LR) among squirrel groups for the premaxillofrontal (Fig 3B; p < 0.001, F_4,54_ = 6.81), maxillofrontal (Fig 3D; p = 0.0027, F_4,53_ = 4.64), and sagittal sutures (Fig 3F; p < 0.0001, F_4,50_ = 10.20), but no differences for either the nasofrontal (Fig 3C; p = 0.182, F_4, 54_ = 1.62) or coronal sutures (Fig 3E; p = 0.223, F_4, 53_ = 1.48). Our post-hoc pairwise comparisons demonstrate significant differences between allopatric red squirrels and allopatric Douglas squirrels for the premaxillofrontal (p = 0.0041), maxillofrontal (p = 0.0021), and sagittal (p = 0.0002) sutures. We also find significant pairwise differences between the hybrid-zone red squirrels and the hybrid-zone Douglas squirrels for the premaxillofrontal (p = 0.0013), maxillofrontal (p = 0.037), and sagittal (p = 0.01) sutures. Hybrids are only significantly different from the hybrid-zone Douglas squirrels in their premaxillofrontal (p = 0.0047) and sagittal sutures (p = 0.028), but not in their maxillofrontal suture (p = 0.36). Hybrids are not significantly different from hybrid-zone red squirrels in any of their sutures.

The first two axes of the PCA of all five sutures, PC1 and PC2, explain 65.95% of the variation in suture complexity (Fig 3A; PC1: 45.95%; PC2: 20.00%). PC1 is primarily driven by the complexity of the premaxillofrontal, maxillofrontal, and sagittal sutures with negative PC scores representing greater complexity for these three sutures. PC2 is primarily determined by variation in coronal and nasofrontal sutures; with increased complexity in the nasofrontal suture associated with positive PC scores, whereas increased complexity in coronal sutures is associated with negative PC scores. An ANCOVA reveals a significant difference in PC1 scores among squirrel groups (Group: p < 0.0001, F_4,45_ = 13.38; sex: p = 0.002, F_1,45_ = 10.47; size: p = 0.42, F_1,45_ = 0.67), but no significant difference is present for PC2 among groups (Group p = 0.528, F_4,45_ = 0.81; sex: p = 0.97, F_1,45_ = 0.002; size: p = 0.38, F_1,45_ = 0.80). Post-hoc tests reveal a significant difference in PC1 scores between Douglas squirrels and red squirrels in allopatry (p = 0.0002), as well as in sympatry (p = 0.013), however, no significant differences in PC1 scores between allopatric Douglas squirrel and hybrid-zone Douglas squirrels (p = 0.73) or between allopatric red squirrels and hybrid-zone red squirrels (p = 0.84). Hybrids differ significantly in PC1 scores from hybrid-zone Douglas squirrels (p = 0.0379), but not from hybrid-zone red squirrels (p = 0.386).

### Geometric morphometrics

**Fig 4:**
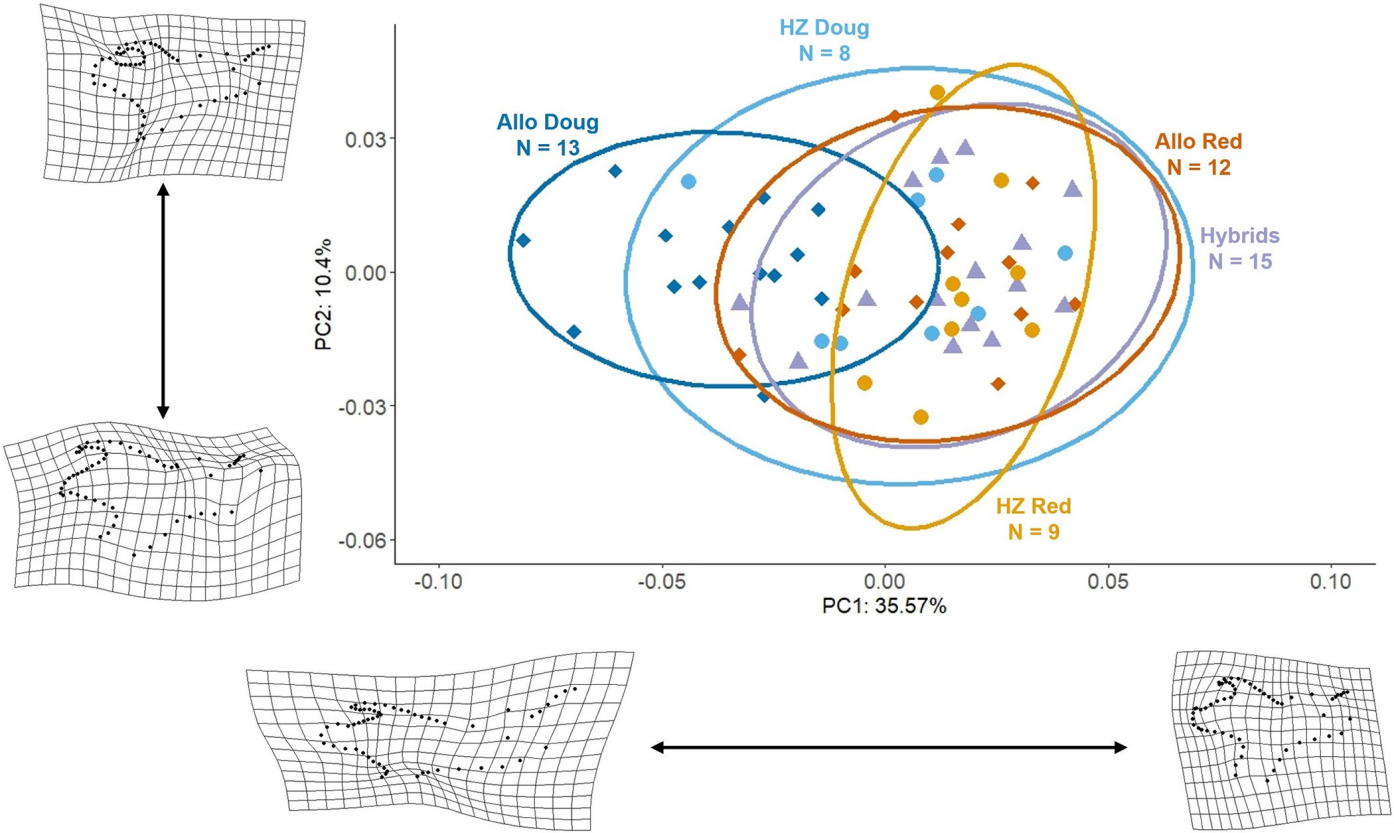
Principal component analysis of mandible shape in lateral view. Deformation grids for principal component axes accompany their respective axis and were based on the graphed extremes for the figure. Abbreviations are provided in Fig 2.

Monte Carlo randomization supported PC1 (p < 0.001) and PC2 (p < 0.001) as significant axes for the morphospace PCA, with all other axes being nonsignificant (p > 0.99); only the first two axes were retained for subsequent analyses. PC1 and PC2 explain 45.97% of the variation in mandible morphology among the squirrels studied (Fig 4; PC1: 35.57%; PC2: 10.40%). Specimens with more positive PC1 values have an anteroposteriorly shortened mandible, a dorsoventrally shortened condylar process, an dorsoventrally heightened enlarged angular process, and a higher coronoid process. Positive PC2 values are associated with a short ramus, including a reduced coronoid process and a short angular process.

**Fig 5:**
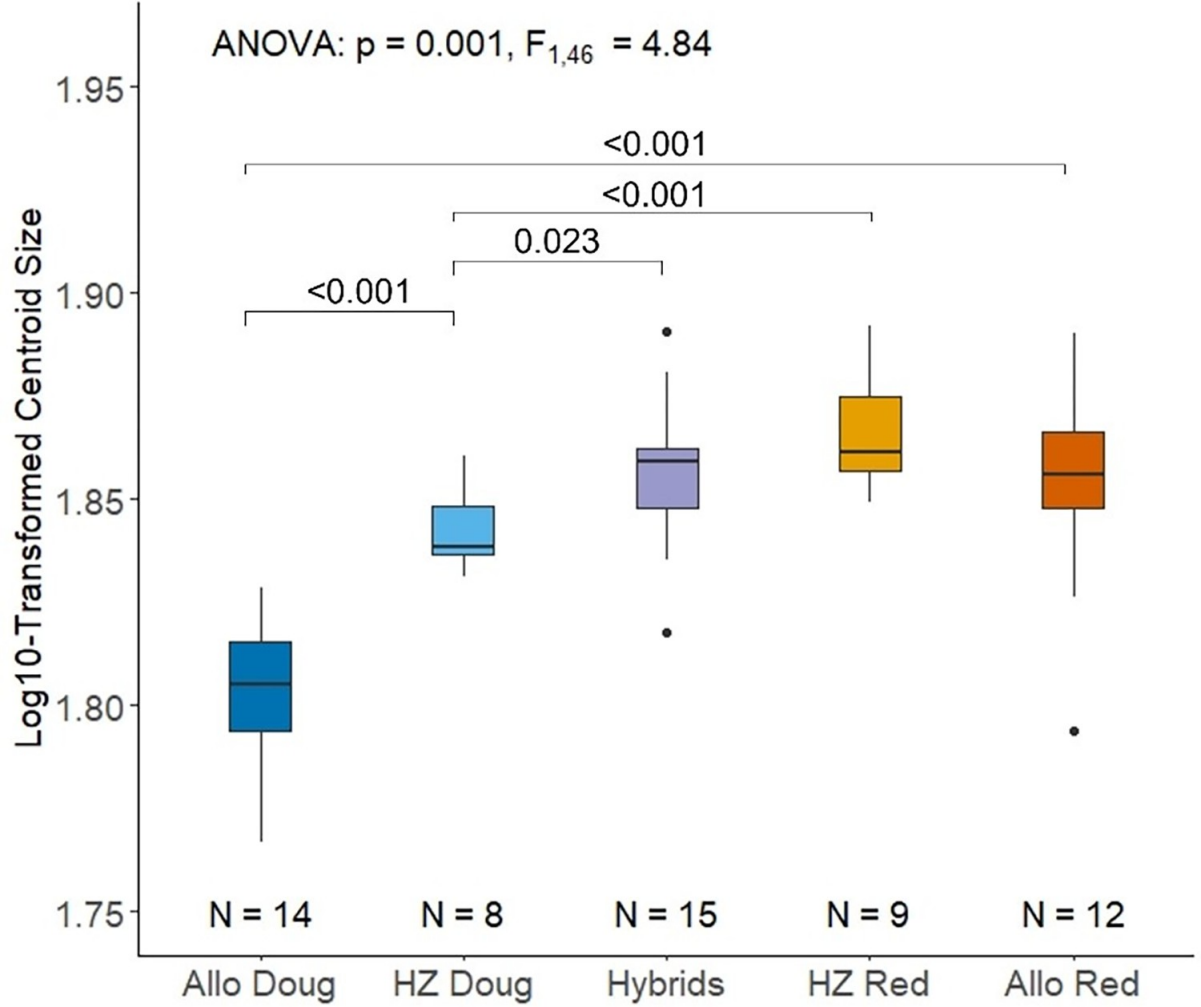
Variation in log10-transformed mandible centroid size across squirrel groups. Values on comparison bars represent significant p-values from pairwise comparisons. Outliers are indicated by black dots. Abbreviations are provided in Fig 2.

We analyzed the possible effects of centroid size and sex on mandible shape to ascertain the roles of allometric scaling and sexual dimorphism. The Procrustes ANOVA of PC1 and PC2 showed that mandible shape is significantly correlated with log10-transformed centroid size (Table 1; p = 0.001, F_1,46_ = 5.19) and the interaction between group and centroid size (p = 0.007, F_4,46_ = 2.38); but mandible shape was not significantly affected by sex (p = 0.264, F_1,46_ = 0.61) or interactions between sex and size (p = 0.166, F_1,46_ = 0.99). The ANOVA of centroid size demonstrated a significant difference in size among the different squirrel groups (Fig 5; p < 0.001, F_4,53_ = 22.94). Post-hoc comparisons indicate significant differences in centroid size between allopatric red squirrels and allopatric Douglas squirrels (p < 0.0001), as well as between hybrid-zone red squirrels and hybrid-zone Douglas squirrels (p = 0.0011). In addition, hybrid-zone Douglas squirrels are significantly larger than allopatric Douglas squirrels (p<0.0001). There are no significant differences between hybrid-zone red squirrels and allopatric red squirrels (p = 0.498). Hybrids are significantly larger than hybrid-zone Douglas squirrels (p = 0.022) but are not different from hybrid-zone red squirrels (p = 0.13). The Procrustes ANOVA indicates a significant effect of group on mandible shape (p = 0.001, F_4,46_ = 6.41); however, there were no significant pairwise differences associated with Procrustes data after correction for multiple testing.

**Table 1:** Procrustes ANOVA of lateral view mandible shape data. Relationships assessed were for squirrel group, sex, log transformed centroid size, and the interactions size by group and size by sex. Significant p-values are shown in bold.

	Df	SS	MS	Rsq	F	Z	Pr(>F)
Group	4	0.002426	0.000606	0.38768	13.3115	6.4123	**0.001**
Sex	1	5.74E-05	5.74E-05	0.00918	1.2603	0.6071	0.264
Size	1	0.001139	0.001139	0.18198	24.9946	5.1933	**0.001**
Group:Size	4	0.000455	0.000114	0.07271	2.4967	2.3804	**0.007**
Sex:Size	1	8.47E-05	8.47E-05	0.01353	1.8587	0.9875	0.166
Residuals	46	0.002095	4.56E-05	0.33492			
Total	57	0.006257					

**Fig 6:**
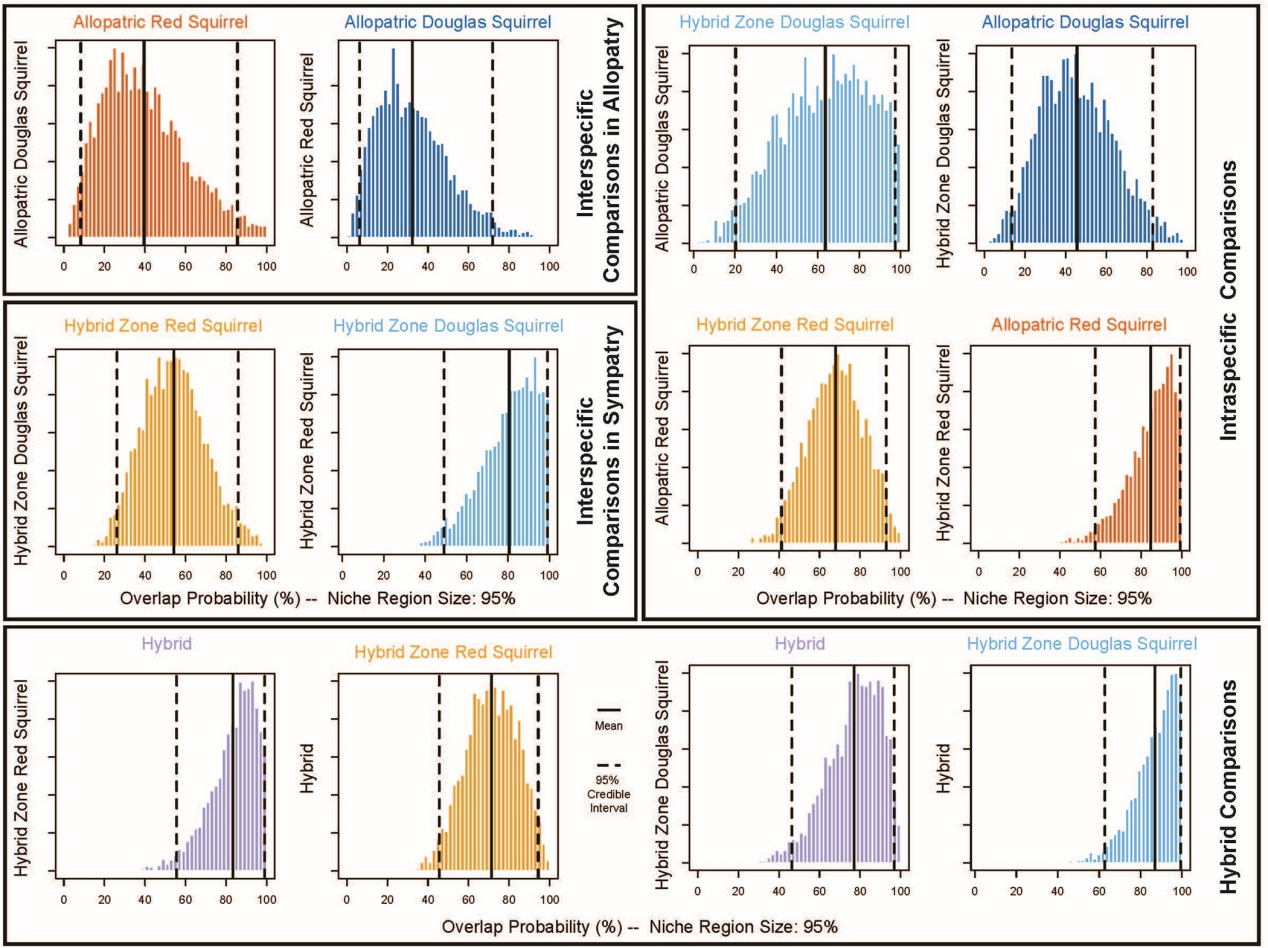
The probability of mandible morphospace overlap between squirrel groups for geometric morphometric principal components 1 and 2 representing shape and log10-transformed centroid size representing size. Data shown are a posterior histogram of 3000 iterations of overlap metric calculations, whereby the x-axis of each pairwise comparison is the range of overlap probabilities in percentage, and the y-axis is the frequency of that probability having been observed in the calculation. Mean overlap probability is shown as a solid black line, and 95% confidence intervals are shown as dashed lines for all pairs. The plots shown here represent key comparisons for this study; additional comparisons are shown in S1 Fig.

The analysis of morphospace overlap using nicheROVER reveals varying degrees of overlap in mandible shape and size among squirrel groups (Fig 6; S1 Fig). Allopatric Douglas squirrels show a high degree of overlap with hybrid-zone Douglas squirrels but low overlap with allopatric red squirrels, hybrid-zone red squirrels, and hybrids. Hybrid-zone Douglas squirrels show a high degree of overlap with hybrids and a greater probability of overlap with red squirrel populations than with Douglas squirrels in the parental range. Hybrids have a strong probability of overlapping with all other squirrel groups except for allopatric Douglas squirrels. Finally, both allopatric red squirrels and hybrid-zone red squirrels have a high overlap probability with other squirrel groups, except allopatric Douglas squirrels.

### Hybrid admixture regressions

A minimum admixture score of 0.18 and maximum admixture score of 0.83 were calculated for the hybrids studied, with a mean score of 0.5353 (± 0.0749 95% CI) for the 19 hybrid individuals. Regressions of admixture proportion for hybrids and their measured dietary ecomorphological traits were tested for genetic correlations. Admixture proportions (Q) were not significantly correlated with BFQ (R^2^ = 0.01, p = 0.771) or any of the five LR measurements (nasofrontal: R^2^ = 0.01, p = 0.627; premaxillofrontal: R^2^ = 0.001, p = 0.879; maxillofrontal: R^2^ = 0.02, p = 0.558; coronal: R^2^ = 0.04, p = 0.464; sagittal: R^2^ = 0.03, p = 0.468). Admixture was also not significantly correlated with PC1 of the morphospace PCA (R^2^ = 0.08, p = 0.314) or centroid size (R^2^ = 0.004, p = 0.811).

## Discussion

The dietary ecomorphology of an organism is phenotypically complex, involving multiple integrated morphological traits that enable interactions with the environment [53–57]. This is especially relevant for mammal skulls, where food acquisition and processing are determined mainly by muscle attachments, muscle masses, and mechanical levers that control bite force and, therefore, the food items that can be accessed [58–60]. In this study, we investigated the evolution of three morphological traits associated with feeding, including incisor shape as a measure of bite force, cranial suture complexity, and mandible shape. We found a consistent pattern across all morphological traits that red squirrels have traits for stronger BFQ than Douglas squirrels in both allopatry and sympatry, as well as a tendency for the hybrids to more closely resemble red squirrels than Douglas squirrels in morphology.

### Inter-specific ecomorphological differences in allopatry

We find support for our hypothesis that ecomorphological divergence between allopatric red squirrels and Douglas squirrels is consistent with expectations under strong ecological divergent selection; this is true for all morphological traits studied. Red squirrels possess a stronger bite force (as measured by their incisor strength index), as well as greater suture complexity than Douglas squirrels. In these allopatric populations, it has been previously documented that red squirrels possess a prominent sagittal crest, associated with an expectedly larger temporalis muscle for stronger gnawing of hard conifer cones, whereas Douglas squirrels display a lower sagittal “plateau” [43, 45]. The sagittal crest forms along the sagittal suture. Thus, our findings of greater sagittal suture complexity in red squirrels are consistent with their stronger temporalis muscles exerting greater forces to this suture. The relationship between greater biting forces, the sagittal suture, and sagittal crest size could be addressed in future studies accompanied by temporal muscle dissections to quantify muscle attachment space. Furthermore, we also find greater complexity in the premaxillofrontal and maxillofrontal sutures in red squirrels. Greater cranial suture complexity is expected to be important in alleviating the forces associated with a strong biting action [31–35]. These specific sutures are associated with tooth-bearing bones (premaxilla and maxilla) and are specifically expected to be more complex in response to increased masticatory strains [61]. The stronger biting ability of red squirrels is also apparent in their mandible shape. Allopatric red squirrels have a shorter mandibular corpus, a dorsoventrally shorter condylar process, a dorsoventrally longer angular process, and a higher coronoid process than allopatric Douglas squirrels. This morphological shape in red squirrels enables a relatively greater mechanical advantage. This pattern is consistent with the general pattern defining nut-eating squirrels in which they have been characterized as having a robust mandibular corpus, high ramus, and well-developed angular and coronoid processes [39]. Thus, the foundation of morphological divergence between allopatric red squirrels and Douglas squirrels appears to be occurring in the components of the mandible most directly associated with a durophagous diet. Our findings of ecomorphological divergence in the craniomandibular shape between *Tamiasciurus* species are interesting in the context of skull evolution in squirrels, which have skulls that are generally considered to be extreme examples of morphological conservation [28, 39, 62–65].

The morphological differences in red squirrels that enable stronger bite force is consistent with the greater hardness of their primary food item. Seeds from lodgepole pine are a primary food item for red squirrels east of the Cascade Range. The dry climate in this region allows for high-intensity fires that threaten forest establishment. As a consequence, lodgepole pine has evolved the ability to produce serotinous (closed) cones that only open following forest fires to protect their seeds during fires. This trait is variable albeit important for lodgepole pine throughout the Rocky Mountain region, including the eastern Cascade Range (pers. comm.). Serotinous cones are harder in texture and have reduced seed numbers than non-serotinous cones [66–68]. However, the hard lodgepole pinecone creates an important selective pressure on red squirrels to evolve greater biting strength to access the seeds from the cones [46]. In contrast, Douglas squirrels live in the mesic coastal forests west of the Cascade Range, where lodgepole pine is rare or nonexistent and where serotiny is not a seed preservation strategy for any conifer species [42]. Thus, Douglas squirrels feed largely on seeds from relatively soft-coned conifer species, such as Douglas fir *(Pseudotsuga menziesii)*, that do not require biting strengths that are as demanding.

Our study reveals an interesting difference in morphological variation for both suture complexity and mandible shape between these *Tamiasciurus* species. Red squirrels show greater variation in both morphospace and suture complexity than Douglas squirrels, indicating their greater range of phenotypic variation in dietary ecomorphology. Morphology can become more variable along an adaptive plateau rather than a sharp adaptive peak, contributing to such signals of greater variation [65, 69]. The greater range of morphological variation in red squirrels and hybrids than in Douglas squirrels may reflect a wider adaptive plateau and a greater range of viable phenotypes for suture complexity and mandible shape.

### Inter-specific ecomorphological differences in sympatry within the hybrid zone

Where red squirrels and Douglas squirrels are sympatric within the hybrid zone, we find ecomorphological divergence patterns similar to the patterns observed in allopatry outside the hybrid zone. Indeed, despite competing for the same food resources and experiencing the same dietary biomechanical needs within the hybrid zone, red squirrels maintain greater bite force and suture complexity than Douglas squirrels. The persistence of morphological differences in sympatry suggests that divergence in the genetic architecture of some craniomandibular traits is maintained. This occurs despite potential homogenization effects of backcrossed hybridization, introgression of genetic variation, and perhaps some limitations in the role of phenotypic plasticity allowing convergent morphologies in a shared environment. Also of note is the decrease in bite force for both species within the hybrid zone when compared with their conspecifics outside of the hybrid zone. This may suggest weaker biomechanical demands presented by conifer cones within the hybrid zone. This is potentially due to the hybrid zone containing both large tracts of subalpine forests that are dominated by the soft-coned Engelmann spruce (*Picea engelmannii*), as well as a unique transitional forest that contains a mixture of both mesic and dry conifer species [70]. These findings, however, contrast with our findings on mandibular morphospace, as both species display similar mandible shapes. We propose that plasticity may have a more critical role in these morphological traits and allows for greater convergence in muscle attachment and mechanical levers in a shared environment between these two squirrel species. Our findings add to previous studies in the *Tamiasciurus* hybrid zone that niche partitioning between red squirrels and Douglas squirrels is non-existent [45, 51].

Our findings also do not support expectations of the ecological competitive exclusion theory that species with shared resource requirements cannot stably coexist in an environment [71, 72]. Character displacement is one mechanism that allows sympatric species to reduce competition and coexist [8, 73–75], as shown in the feeding apparatus of other species in secondary contact [27]. However, our study does not show evidence for character displacement because the ecomorphological divergence in sympatry between the two squirrel species is not greater than the differences between them in allopatry. This finding is interesting in that these species are known to compete for territory access in sympatry [42, 51]. Interestingly, rather than convergence or character displacement, we find reduced bite force strength in both Douglas squirrels and red squirrels within the hybrid zone compared to their respective allopatric populations. However, the differences in bite force strength between Douglas squirrels and red squirrels remained relatively similar, suggesting that niche partitioning may occur in the hybrid zone, contrary to existing hypotheses for this system [45]. Despite the persistence of differences between Douglas squirrels and red squirrels within the hybrid zone, we also observe that both species had lower bite force in the sympatric hybrid zone than in allopatry. For red squirrels, this shift may be associated with plasticity, as we would expect that they experience a softer diet in the hybrid zone than the allopatric range; however, we would expect the dietary challenge in the hybrid zone to be greater for Douglas squirrel compared to their allopatric range, yet they demonstrated a decline in bite force. The cause of this shared decline in bite force within the hybrid zone merits exploration in future studies. Another consideration for the lack of character displacement between sympatric red squirrels and Douglas squirrels is the presence of viable hybrids. Hybridization can have a dampening effect on character displacement between co-occurring and competing species where hybrids are viable and possess intermediate phenotypes [52]. However, this is not consistent with the hybrid phenotypes observed here, as hybrids phenotypically mirrored red squirrels rather than being intermediate between the parental species.

### Impact of hybridization on ecomorphologies

We find that ecomorphologies of hybrid squirrels follow different general expectations for hybrid phenotypes, including phenotypes that are more like one of the parental species, as well as phenotypes that span across the range of phenotypic variation of both parental species. BFQ, sagittal suture, and premaxillofrontal sutures in hybrids are more like sympatric red squirrels than Douglas squirrels. One explanation for why hybrid morphologies resemble red squirrel morphologies more closely and have greater morphological variation than Douglas squirrel is the genetic dominance of red squirrel alleles underlying these morphological traits. This is consistent with the lack of correlation between genetic ancestry proportions in hybrids and their incisor strength index or their suture complexity, suggesting further evidence that non-additive genetic variation, such as dominance or environmental effects, contributes to hybrid morphologies. However, a confounding factor that can also produce this pattern is the ecological selection for hybrid morphologies that more closely resemble red squirrel morphologies. Additional studies are needed to distinguish between the effects of genetic effects and selective advantages of red squirrel phenotypes. Addressing this possibility of competition may also shed light on the seemingly stable coexistence of the co-occurring species within the hybrid zone.

In contrast to the incisor strength index and the sagittal and premaxillofrontal sutures, the mandible shapes of hybrids and parental species located within the hybrid zone greatly overlap. One possibility for the similarity in mandible shape between hybrids and both parental species is that plasticity may have a more substantial effect on bony structures of the mandible than tooth shape [76]. Mandibles can be remodeled by their constant interactions with muscles and surrounding tissues, particularly when food hardnesses are variable between populations [23, 77]. In the *Tamiasciurus* hybrid zone, hybrids and parental species are found in syntopy and gnaw on cones from the same conifer species [42, 51]. Thus, it is conceivable that the shared biomechanical demands on the mandibles of both squirrel species and their hybrids in the hybrid zone, combined with the plasticity of the mandible have led to the overlap in mandibular shape.

Biologists have spent considerable effort in studying both the evolution of craniomandibular ecomorphogical traits, as well as hybrid zones across different mammalian taxa [78–82]. However, very few studies have combined these areas of research in trying to understand the consequences of admixture on trophic morphologies in mammals. One exception is the well-studied hybrid zone in central Europe between subspecies of house mice (*Mus musculus domesticus and M*.*m*.*musculus)* [79]. The phenotypic consequences of hybridization include heterosis, enhanced hybrid phenotypes, of the mandible and skull in hybrids [23, 83]. Despite the impressive breadth of research on craniomandibular traits in house mice hybrids, including developmental stability, genetic architecture, and gross morphology [24, 84–86], existing studies are mostly laboratory-based and not necessarily true to ecology. In addition, the similar generalist diets of both mice subspecies can make it challenging to interpret the adaptive significance of morphological divergence. Our study system on *Tamiasciurus* squirrels presents contrasting virtues, and drawbacks to the house mice system as the adaptive significance of morphological variation are more apparently linked to the divergent ecologies between the two squirrel species. However, the larger body size of the squirrels and lower fecundity limit the types of experimental studies that can be conducted to thoroughly understand the developmental and genetic aspects of craniomandibular morphology.

Divergent ecological selection, interspecific competition, and hybridization are all ecological processes that can drive resource partitioning between closely related species and, ultimately, divergence in ecomorphologies. Our findings suggest that divergent selection associated with dietary differences in cone hardness has been an important driver of ecomorphological divergence between *T*. *douglasii* (soft-cone consumer) and *T*. *hudsonicus* (hard-cone consumer). Further experimental work, including dietary manipulation, is needed to examine the importance of plasticity. Through our study, we show that significant differences in ecomorphological traits are still observable in closely related taxa within a taxonomic group, like sciurids, that show a high degree of morphological conservatism. Finally, our use of fine-scale morphological data, such as bite force and suture complexity, provides additional evidence that high-resolution continuous traits are preferable over coarse traits for correctly detecting divergence instead of convergence [87].

## Materials and methods

### Specimen selection

To assess morphological differences between red squirrels, Douglas squirrels, and hybrids, we used a sample of 70 museum specimens across five groups (S1 Table): allopatric red squirrels (n = 14), hybrid zone red squirrels (n = 13), hybrids (n = 19), hybrid zone Douglas squirrels (n = 10), and allopatric Douglas squirrels (n = 14). Some specimens had varying degrees of damage and were excluded from some analyses. Final sample numbers for analyses are shown in the respective figures for those analyses. All specimens in this study are housed at the Burke Museum of Natural History (S1 Table; see S2 Fig for representative examples). Allopatric specimens were selected such that they were outside the hybrid zone (see S1 Table; Fig 1). The selection of specimens from within the hybrid zone was based on previous studies of the *Tamiasciurus* hybrid zone [42, 43]. We also used previously published estimates of admixture proportions for these same specimens [43, 51] based on the program STRUCTURE, an unsupervised clustering method implemented in a Bayesian framework. The estimated individual admixture proportions (Q) represent the fraction of an individual’s genome coming from a given population [88] and were used to assign individuals to different genotypic classes (following assignment classes of [89]). We assigned individuals as either pure red squirrel (Q≥ 0.90), pure Douglas squirrel (Q ≤ 0.10), or hybrid (0.10 < Q < 0.90).

### Bite force estimation

**Fig 7:**
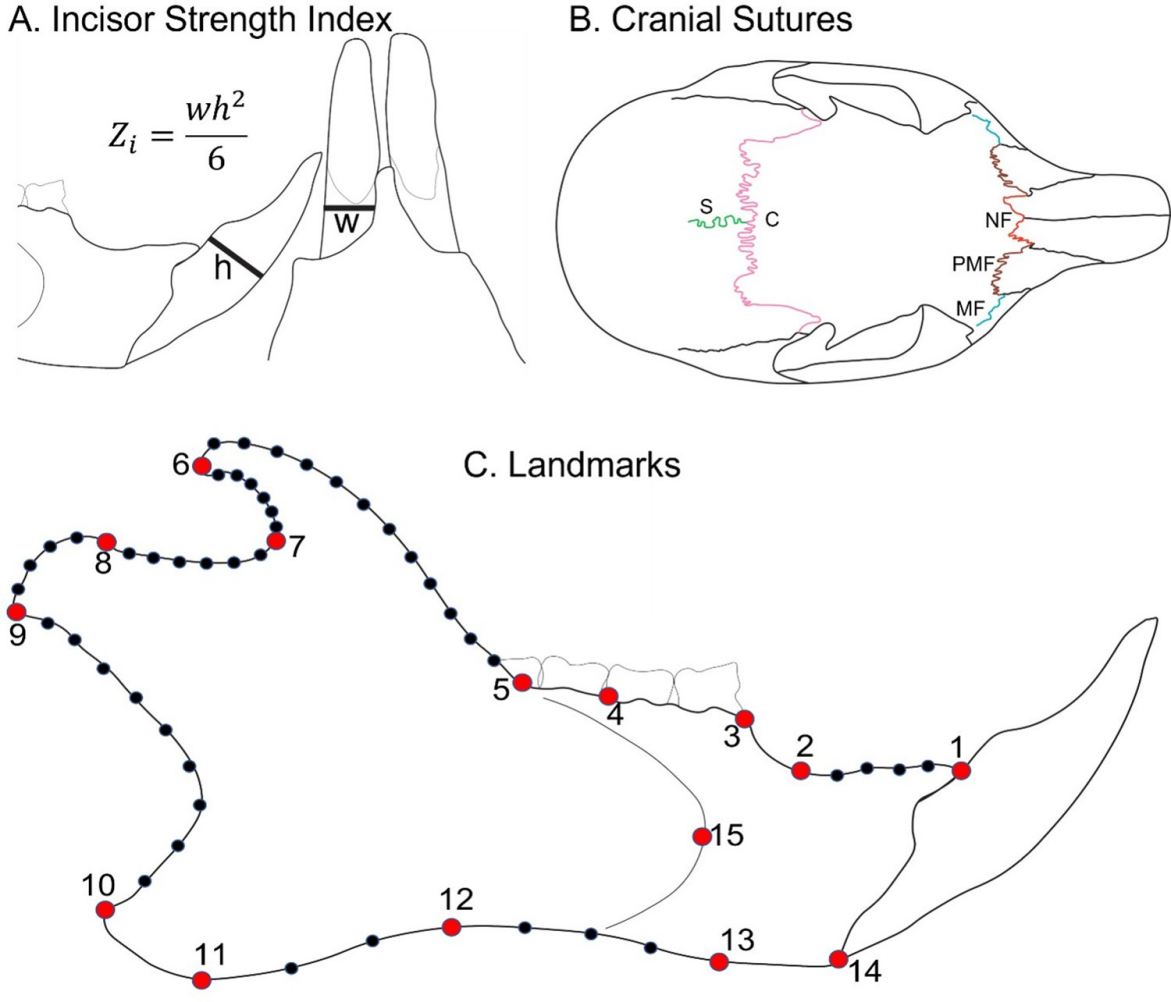
(A) Measurements of incisors in lateral and occlusal views and equation of the incisor strength index (labeled Zi) used as a proxy for bite force. (B) Schematic of cranial sutures used in this study with the nasofrontal suture (labeled NF) in red, the premaxillofrontal suture (labeled PMF) in brown, the maxillofrontal suture (labeled MF) in yellow, the coronal suture (labeled C) in pink, and the sagittal suture (S) in green. (C) Position of landmarks (large red circles) and semilandmarks (small black circles) on the mandible in lateral view. Descriptions of landmarks and semilandmarks are provided in S2 and S3 Tables.

Maximal bite force was estimated from complete incisors using an incisor strength index (Z_i_) of Z_i_ = ((antero-posterior length of incisor)^2^ x (medio-lateral width of incisor))/6 (as detailed in [27]. This measure is strongly correlated with maximum *in vivo* bite force across Rodentia ((log_10_(bite force) = 0.566 log_10_ (Z_i_) + 1.432, R^2^ = 0.956, [27]), and can be used to estimate maximal bite force for museum specimens where *in vivo* bite force data are unavailable. The anteroposterior length and the medio-lateral width of the lower right incisor of each specimen was measured using a Dino-Lite Edge AM7915 digital microscope by a single investigator (DMP) (Fig 7A). Because bite force scales allometrically with body size [29, 30, 90], bite force was converted to a bite force quotient (BFQ) using the residuals of the regression between log_10_(bite force) and log_10_(body mass) for all squirrel specimens in this study (following the method of [29]), with the regression ((log_10_(bite force) = 0.3849 log_10_ (body mass) + 0.6970, R^2^ = 0.419)) for this dataset. Bite force data were analyzed with an ANCOVA (using sex as a covariate) in RStudio version 1.4.1103 [91]. When ANCOVA results were significant, post-hoc pairwise comparisons were performed to test for differences among squirrel groups. To test for the potential effects of hybrid admixture on bite force estimation, we used a linear least squares regression of bite force and admixture score. We predicted that (1) red squirrels have a stronger BFQ than Douglas squirrels when compared allopatrically, (2) have more similar BFQ when compared sympatrically, and (3) hybrids have a phenotype intermediate between the sympatric parental species.

### Suture morphology

We quantified a complementary measure of bite force and skull suture complexity for all museum specimens with uninterrupted sutures of intact skulls. We measured five suture lines (coronal, sagittal, nasofrontal, maxillofrontal, and premaxillofrontal sutures; Fig 7B) for each specimen. To do this, we photographed the dorsal view of the cranium from a fixed distance using a Canon EOS Rebel T6i. We then made vector graphics of sutures in Inkscape v0.93 (inkscape.org) by tracing the total length of the path of each suture on the images. We chose the right side of the crania to measure the maxillofrontal and premaxillofrontal sutures unless these were damaged, in which case, we measured the sutures on the left side. The complexity of each suture was quantified as a length ratio (LR), whereby the total length of the suture path is divided by the linear distance between the start and end [35, 92, 93]. Suture complexity data were analyzed with an ANCOVA, with sex as a covariate. When ANCOVA results were significant, post-hoc pairwise comparisons were performed to test for differences among squirrel groups. To test for a possible multivariate effect of suture complexity, all five sutures were also analyzed together using a principal component analysis (PCA). We used ANOVAs to test differences in PC1 and PC2 scores among squirrel groups. Finally, to test for the potential effects of hybrid admixture on suture complexity, we performed linear least squares regressions of suture complexity and admixture score for all five sutures. We predicted that (1) red squirrels have more complex sutures than Douglas squirrels when compared allopatrically, (2) have similarly complex sutures when compared sympatrically, and (3) hybrids have a phenotype intermediate between the sympatric parental species.

### Geometric morphometrics

To test for morphological divergence and morphospace overlap among the squirrel groups studied, we performed a geometric morphometric analysis of lower jaw shape including all complete specimens. The mandibles were photographed in lateral view on the same plane at a fixed distance using a Canon EOS Rebel T6i camera. For each specimen, 15 landmarks and 45 semilandmarks were digitized in TpsDig2 software [94]. The landmarks scheme was adapted from schemes used for squirrels [39] and hybridizing mice [24]. The semilandmarks used are equidistant points resampled to the lowest number, properly encapsulating the shape of the curve from homologous curves (Fig 7C, Landmark Description in S2 and S3 Tables). Landmark data were imported and analyzed using the R package geomorph v. 4.0.1 [95]. Semilandmarks were registered as sliders and slid according to the bending energy criterion [96]. Landmarks and semilandmarks were subjected to Generalized Procrustes Analysis (GPA) [97, 98]. A PCA was performed on the GPA data to explore group differences in shape data. A Monte Carlo randomization test run in biostats [99] was used to assess axes for which the amount of variation represented is significantly different from the null hypothesis. Procrustes ANOVAs were performed on these data in geomorph to assess the relationships between shape, size, sex, and group, with size represented by the log10-transformed centroid sizes from GPA. Pairwise comparisons were generated from these data in geomorph. We predicted that allopatric red squirrels would occupy a distinct morphospace from allopatric Douglas squirrels, that sympatric red squirrels and Douglas squirrels would have similar morphospace, and hybrids occupy an intermediate, and potentially overlapping, morphospace between the parental species.

Morphospace overlap between groups was analyzed using nicheROVER [100] using PC1 and PC2 scores, which represent the shape, and log10-transformed centroid size, which represents size. This analysis provides the probabilities of overlap between squirrel group pairs by taking advantage of a method developed to analyze multidimensional niche space overlap [101, 102]. Distributions were generated from the posterior distributions of 3,000 permutations of overlap probability. We predicted that for each species, the allopatric squirrels would have a high probability of overlap with hybrid zone conspecifics but low overlap with heterospecific populations in allopatry, whereas hybrids would have a moderate to high probability of overlap with the other four squirrel groups. To test for the potential effects of hybrid admixture on mandible shape, we used a linear least squares regression of bite force and admixture score.

## Supporting information

S1 Fig Analysis of overlap probability of mandible geometric morphometric principal components 1 and 2 representing shape and log10-transformed centroid size representing size. Data shown are a posterior histogram of 3000 iterations of overlap metric calculations, whereby the x-axis of each pairwise comparison is the range of overlap probabilities in percentage and the y-axis is frequency of that probability having been observed in calculation. Comparisons are for morphospace overlap between the squirrel group in the grid row on the squirrel group in the grid column. Mean overlap probability is shown as a solid black line and 95% confidence intervals are shown as dashed lines for all pairs.
(DOCX)

S2 Fig Representative examples for hybrid zone specimens. (A) Douglas squirrel cranium from specimen UWBM 20809. (B) Douglas squirrel left mandible from specimen UWBM 20809, image is flipped. (C) Hybrid squirrel cranium from specimen UWBM 21078. (D) Hybrid squirrel right mandible from specimen UWBM 21078. (E) Red squirrel cranium from specimen UWBM 21034. (F) Red squirrel left mandible from specimen UWBM 21034, image is flipped.
(DOCX)

S1 Table Specimen museum IDs and locations. (XLSX)

S2 Table Homologous landmark descriptions. (DOCX)

S3 Table Semilandmark assignment and descriptions. (DOCX)

## References

[1] Jones K, Law CJ. Differentiation of craniomandibular morphology in two sympatric Peromyscus mice (Cricetidae: Rodentia). Mammal Res. 2018; 63: 277–283. doi: 10.1007/s13364-018-0364-2

[2] Feller AF, Selz OM, McGee MD, Meier JI, Mwaiko S, Seehausen O. Rapid generation of ecologically relevant behavioral novelty in experimental cichlid hybrids. Ecol Evol. 2020; 10: 7445–7462. doi: 10.1002/ece3.6471 32760540 PMC7391563

[3] Selz OM, Seehausen O. Interspecific hybridization can generate functional novelty in cichlid fish. Proc Royal Soc B. 2019; 286: 20191621. doi: 10.1098/rspb.2019.1621 31640510 PMC6834035

[4] Nürnberger B, Barton N, MacCallum C, Gilchrist J, Appleby M. Natural selection on quantitative traits in the *Bombina* hybrid zone. Evolution. 1995; 49: 1224–1238. doi: 10.2307/2410447 28568525

[5] Milano ER, Kenney AM, Juenger TE. Adaptive differentiation in floral traits in the presence of high gene flow in scarlet gilia (*Ipomopsis aggregata*). Mol Ecol. 2016; 25: 5862–5875. doi: 10.1111/mec.13752 27392816

[6] Knief U, Bossu CM, Saino N, Hansson B, Poelstra J, Vijay N, et al. Epistatic mutations under divergent selection govern phenotypic variation in the crow hybrid zone. Nat Ecol Evol. 2019; 3: 570–576. doi: 10.1038/s41559-019-0847-9 30911146 PMC6445362

[7] Pfennig KS, Pfennig DW. Character displacement: ecological and reproductive responses to a common evolutionary problem. Q Rev Biol. 2009; 84: 253–276. doi: 10.1086/605079 19764283 PMC3279117

[8] Pfennig DW, Pfennig PF. Character displacement and the origins of diversity. Am Nat. 2010; 176: S26–S44. doi: 10.1086/657056 21043778 PMC3285564

[9] Reifová R Reif J, Antczak M, Nachman MW. Ecological character displacement in the face of geneflow: evidence from two species of nightingales. BMC Evol Biol. 2011; 11: 138. doi: 10.1186/1471-2148-11-138 21609448 PMC3121626

[10] Cody ML. Convergent characteristics in sympatric species—a possible relation to interspecific competition and aggression. Condor. 1969; 71: 223–239. doi: 10.2307/1366300

[11] Cody ML. Character convergence. Annu Rev Ecol Evol Syst. 1973; 4: 189–211. doi: 10.1146/annurev.es.04.110173.001201

[12] Leary CJ. Evidence of convergent character displacement in release vocalizations of *Bufo fowleri* and *Bufo terrestris* (Anura; Bufonidae). Anim Behav. 2001; 61: 431–438. doi: 10.1006/anbe.2000.1597

[13] Parsons KJ, Son YH, Albertson CR. Hybridization promotes evolvability in African cichlids: Connections between transgressive segregation and phenotypic integration. Evol Biol. 2011; 38: 306–315. doi: 10.1007/s11692-011-9126-7

[14] Selz OM, Lucek K, Young KA, Seehausen O. Relaxed trait covariance in interspecific cichlid hybrids predicts morphological diversity in adaptive radiations. J Evol Biol. 2014; 27: 11–24. doi: 10.1111/jeb.12283 24330234

[15] Wilde GR, Echelle AA. Morphological variation in intergrade pupfish populations from the Pecos River, Texas, U.S.A. J Fish Biol. 1997; 50: 523–539. doi: 10.1111/j.1095-8649.1997.tb01947.x

[16] Loy A, Capula M, Palombi A, Capanna E. Genetic and morphometric evidence of introgression between two species of moles (Insectivora: *Talpa europaea* and *Talpa romana*) in central Italy. J Zool. 2001; 254: 229–238. doi: 10.1017/S0952836901000747

[17] Klingenberg CP, Leamy LJ, Routman EJ, Cheverud JM. Genetic architecture of mandible shape in mice: effects of quantitative trait loci analyzed by geometric morphometrics. Genetics. 2001; 157: 785–802. doi: 10.1093/genetics/157.2.785 11156997 PMC1461535

[18] Klingenberg CP, Leamy LJ, Cheverud JM. Integration and modularity of quantitative trait locus effects on geometric shape in the mouse mandible. Genetics. 2004; 166: 1909–1921. doi: 10.1534/genetics.166.4.1909 15126408 PMC1470826

[19] Workman MS, Leamy LJ, Routman EJ, Cheverud JM. Analysis of quantitative trait locus effects on the size and shape of mandibular molars in mice. Genetics. 2002; 160: 1573–1586. doi: 10.1093/genetics/160.4.1573 11973311 PMC1462040

[20] Monti L, Baylac M, Lalanne-Cassou B. Elliptic Fourier analysis of the form of genitalia in two *Spodoptera* species and their hybrids (Lepidoptera: Noctuidae). Biol J Linn Soc. 2001; 72: 391–400. doi: 10.1111/j.1095-8312.2001.tb01325.x

[21] Valentin A, Sévigny J-M, Chanut J-P. Geometric morphometrics reveals body shape differences between sympatric redfish *Sebastes mentella*, *Sebastes fasciatus* and their hybrids in the Gulf of St Lawrence. J Fish Biol. 2002; 60: 857–875. doi: 10.1111/j.1095-8649.2002.tb02414.x

[22] Nolte AW, Sheets HD. Shape based assignments tests suggest transgressive phenotypes in natural sculpin hybrids (Teleostei, Scorpaeniformes, Cottidae). Front Zool. 2005; 2: 11. doi: 10.1186/1742-9994-2-11 15987531 PMC1198242

[23] Renaud S, Alibert P, Auffray J-C. Mandible shape in hybrid mice. Naturwissenschaften. 2009; 96: 1043–1050. doi: 10.1007/s00114-009-0563-4 19504055

[24] Renaud S, Alibert P, Auffray J-C. Modularity as a source of new morphological variation in the mandible of hybrid mice. BMC Evol Biol. 2012; 12: 141. doi: 10.1186/1471-2148-12-141 22873779 PMC3506452

[25] Konuma J, Sota T, Chiba S. A maladaptive intermediate form: a strong trade-off revealed by hybrids between two forms of a snail feeding beetle. Ecology. 2013; 94: 2638–2644. doi: 10.1890/12-2041.1 24400515

[26] Badyaev AV. The beak of the other finch: Coevolution of genetic covariance structure and developmental modularity during adaptive evolution. Philos Trans R Soc. 2010; 365: 1111–1126. doi: 10.1098/rstb.2009.0285 20194173 PMC2830233

[27] Grant PR, Grant BR. Evolution of character displacement in Darwin’s finches. Science. 2006; 313: 224–226. doi: 10.1126/science.1128374 16840700

[28] Zelditch ML, Ye J, Mitchell JS, Swiderski DL. Rare ecomorphological convergence on a complex adaptive landscape: body size and diet mediate evolution of jaw shape in squirrels (Sciuridae). Evolution. 2017; 71, 633–649. doi: 10.1111/evo.13168 28075012

[29] Wroe S, McHenry C, Thomason J. Bite club: Comparative bite force in big biting mammals and the prediction of predatory behaviour in fossil taxa. Proc Royal Soc B. 2005; 272 (1563): 619–625. doi: 10.1098/rspb.2004.2986 15817436 PMC1564077

[30] Freeman PW, Lemen CA. A simple morphological predictor of bite force in rodents. J Zool. 2008; 275: 418–422. doi: 10.1111/j.1469-7998.2008.00459.x

[31] Herring SW, Mucci RJ. In vivo strain in cranial sutures: The zygomatic arch. J Morphol. 1991; 207: 225–239. doi: 10.1002/jmor.1052070302 1856873 PMC2814820

[32] Herring SW, Teng S. Strain in the braincase and its sutures during function. Am J Phys Anthropol. 2000; 112: 575–593. doi: 10.1002/1096-8644(200008)112:4&lt;575::AID-AJPA10&gt;3.0.CO;2-0 10918130 PMC2813197

[33] Sun Z, Lee E, Herring SW. Cranial sutures and bones: Growth and fusion in relation to masticatory strain. Anat Rec. 2004; 276(2): 150–161. doi: 10.1002/ar.a.20002 14752854 PMC2813868

[34] Moazen M, Curtis N, O’Higgins P, Jones MEH, Evans SE, Fagan MJ. Assessment of the role of sutures in a lizard skull: a computer modeling study. Proc Royal Soc B. 2008; 276: 39–46. doi: 10.1098/rspb.2008.0863 18765341 PMC2614251

[35] Buezas G, Becerra F, Vassallo A. Cranial suture complexity in caviomorph rodents (Rodentia; Ctenohystrica). J Morphol. 2017; 278: 1125–1136. doi: 10.1002/jmor.20699 28480566

[36] Rager L, Hautier L, Forasiepi A, Goswami A, Sanchez-Villagra M. Timing of cranial suture closure in placental mammals: phylogenetic patterns, intraspecific variation, and comparison with marsupials. J Morphol. 2014; 275: 125–140. doi: 10.1002/jmor.20203 24127274

[37] White HE, Goswami A, Tucker AS. The intertwined evolution and development of sutures and cranial morphology. Front Cell Dev Biol. 2021; 9: 690. doi: 10.3389/fcell.2021.653579 33842480 PMC8033035

[38] Ginot S, Herrel A, Claude J, Hautier L. Morphometric models for estimating bite force in *Mus* and *Rattus*: Mandible shape and size perform better than lever-arm ratios. J Exp Biol. 2019. 222: jeb204867. doi: 10.1242/jeb.204867 31097601

[39] Casanovas-Vilar I, van Dam J. Conservatism and adaptability during squirrel radiation: What is mandible shape telling us? PLoS ONE, 2013; 8:e61298. doi: 10.1371/journal.pone.0061298 23593456 PMC3617180

[40] Becerra F, Echeverría AI, Casinos A, Vassallo AI. Another bites the dust: Bite force and ecology in three caviomorph rodents (Rodentia, Hystricognathi). J Exp Zool A Ecol Genet Physiol. 2014; 321: 220–232. doi: 10.1002/jez.1853 24482421

[41] Tanner JB, Zelditch ML, Lundrigan BL, Holekamp KE. Ontogenetic change in skull morphology and mechanical advantage in the spotted hyena (*Crocuta crocuta*). J Morphol. 2010; 271: 353–365. doi: 10.1002/jmor.10802 19862838

[42] Smith CC. The adaptive nature of social organization in the genus of three squirrels *Tamiasciurus*. Ecol Monogr. 1968; 38: 31–63. doi: 10.2307/1948536

[43] Chavez AS, Saltzberg CJ, Kenagy GJ. Genetic and phenotypic variation across a hybrid zone between ecologically divergent tree squirrels (*Tamiasciurus*). Mol Ecol. 2011; 20: 3350–3366. doi: 10.1111/j.1365-294X.2011.05184.x 21771139

[44] Chavez AS, Maher SP, Arbogast BS, Kenagy GJ. Diversification and gene flow in nascent lineages of island and mainland North American tree squirrels (*Tamiasciurus*). Evolution. 2014; 68: 1094–1109. doi: 10.1111/evo.12336 24351034

[45] Smith CC. The indivisible niche of *Tamiasciurus*: an example of nonpartitioning of resources. Ecol Monogr. 1981; 51: 343–363. doi: 10.2307/2937278

[46] Smith CC. The coevolution of pine squirrels (*Tamiasciurus*) and conifers. Ecol Monogr. 1970; 40: 349–371. doi: 10.2307/1942287

[47] Talluto MV, Benkman CW. Conflicting selection from fire and seed predation drives fine-scaled phenotypic variation in a widespread North American conifer. Proc Natl Acad Sci U.S.A., 2014; 111: 9543–9548. doi: 10.1073/pnas.1400944111 24979772 PMC4084486

[48] Benkman CW, Siepielski AM. A keystone selective agent? Pine squirrels and the frequency of serotiny in lodgepole pine. Ecology. 2004; 85: 2082–2087. doi: 10.1890/04-0177

[49] Hall ER. The mammals of North America. John Wiley & Sons, Inc., New York. 1981.

[50] Nagorsen DW. Rodents & lagomorphs of British Columbia. Royal British Columbia Museum, Victoria, BC, Canada. 2005.

[51] Fotis AT, Patel S, Chavez AS. Habitat-based isolating barriers are not strong in the speciation of ecologically divergent squirrels (*Tamiasciurus douglasii* and *T*. *hudsonicus*). Behav Ecol Sociobiol. 2020; 74(3): 1–14. doi: 10.1007/s00265-020-2814-5

[52] Aguilée R, de Becdelièvre B, Lambert A, Claessen D. Under which conditions is character displacement a likely outcome of secondary contact? J Biol Dyn. 2011; 5: 135–146. doi: 10.1080/17513758.2010.491559 22873436

[53] Anderson RA, Macbrayer LD, Herrel A. Bite force in vertebrates: opportunities and caveats for use of a non-pareil whole-animal performance measure. Biol J Linn Soc. 2008; 93: 709–720. doi: 10.1111/j.1095-8312.2007.00905.x

[54] Aguirre LF, Herrel A, van Damme R, Matthysen E. Ecomorphological analysis of trophic niche partitioning in a tropical savanna bat community. Proc Royal Soc B. 2002; 269: 1271–1278. doi: 10.1098/rspb.2002.2011 12065044 PMC1691024

[55] Aguirre LF, Herrel A, van Damme R, Matthysen E. The implications of food hardness for diet in bats. Funct Ecol. 2003; 17: 201–212. doi: 10.1046/j.1365-2435.2003.00721.x

[56] Herrel A, Podos K, Huber SK, Hendry AP. Bite performance and morphology in a population of Darwin’s finches: implications for the evolution of beak shape. Funct Ecol. 2005; 19: 43–48. doi: 10.1111/j.0269-8463.2005.00923.x

[57] Santana SE, Dumont ER, Davis JL. Mechanics of bite force production and its relationship to diet in bats. Funct. Ecol. 2010; 24: 776–784. doi: 10.1111/j.1365-2435.2010.01703.x

[58] Cornette R, Tresset A, Houssin C, Pascal M, Herrel A. Does bite force provide a competitive advantage in shrews? The case of the greater white-toothed shrew. Biol J Linn Soc. 2015; 114: 795–807. doi: 10.1111/bij.12423

[59] Herrel A, Van Damme R, Vanhooydonck B, De Vree E. The implications of bite performance for diet in two species of lacertid lizards. Can J Zool. 2001; 79: 662–670. doi: 10.1139/z01-031

[60] Nogueira MR, Peracchi AL, Monteiro LR. Morphological correlates of bite force and diet in the skull and mandible of phyllostomid bats. Funct Ecol. 2009; 23: 715–723. doi: 10.1111/j.1365-2435.2009.01549.x

[61] Byron C, Segreti M, Hawkinson K, Herman K, Patel S. Dietary material properties shape cranial suture morphology in the mouse calvarium. J Anat. 2018; 233: 807–813. doi: 10.1111/joa.12888 30298923 PMC6231163

[62] Hautier L, Fabre PH, Michaux J. Mandible shape and dwarfism in squirrels (Mammalia, Rodentia): interaction of allometry and adaptation. Naturwissenschaften, 2009; 96: 725–730. doi: 10.1007/s00114-009-0519-8 19288073

[63] Swiderski DL, Zelditch ML. (2010). Morphological diversity despite isometric scaling of lever arms. Evol Biol. 2010; 37: 1–18. doi: 10.1007/s11692-010-9081-8

[64] Zelditch ML, Li J, Tran LAP, Swiderski DL. Relationships of diversity, disparity, and their evolutionary rates in squirrels (Sciuridae). Evolution, 2015; 69: 1284–1300. doi: 10.1111/evo.12642 25787014

[65] Zelditch ML, Li J, Swiderski DL. Stasis of functionally versatile specialists. Evolution, 2020; 74: 1356–1377. doi: 10.1111/evo.13956 32187648

[66] Muir PS, Lotan JE. Disturbance history and serotiny of *pinus contorta* in western Montana. Ecology. 1985; 66: 1658–1668. doi: 10.2307/1938028

[67] Benkman CW, Smith JW, Maier M, Hansen L, Talluto MV. Consistency and variation in phenotypic selection exerted by a community of seed predators. Evolution. 2013; 67: 157–169. doi: 10.1111/j.1558-5646.2012.01736.x 23289569

[68] Parker AL, Benkman CW. Enhanced seed defenses potentially relax selection by seed predators against serotiny in lodgepole pine. Ecol Evol. 2020; 10: 6001–6008. doi: 10.1002/ece3.6339 32607207 PMC7319249

[69] Maestri R, Monteiro LR, Fornel R, Upham NS, Patterson BD, de Freitas TR. The ecology of a continental evolutionary radiation: is the radiation of sigmodontine rodents adaptive? Evolution, 2017; 71: 610–632. doi: 10.1111/evo.13155 28025827

[70] Franklin JF, Dyrness CT. Natural vegetation of Oregon and Washington. Corvallis, OR: Oregon State University Press. 1988.

[71] Gause GF. Experimental analysis of Vito Volterra’s mathematical theory of the struggle for existence. Science, 1934; 79: 16–17. doi: 10.1126/science.79.2036.16-a 17821472

[72] Hardin G. The competitive exclusion principle. Science, 1960; 131: 1292–1297. doi: 10.1126/science.131.3409.1292 14399717

[73] Brown WL, Wilson EO. Character displacement. Syst Zool. 1956; 5: 49–64. doi: 10.2307/2411924

[74] Grant PR. Convergent and divergent character displacement. Biol J Linn Soc. 1972; 4: 39–68. doi: 10.1111/j.1095-8312.1972.tb00690.x

[75] Schluter D. The ecology of adaptive radiation. Oxford University Press. Oxford, U. K. 2000.

[76] Renaud S, Auffray J-C. (2013). The direction of main phenotypic variance as a channel to evolution: cases in murine rodents. Hystrix. 2013; 24: 85–93. doi: 10.4404/hystrix-24.1–6296

[77] Mavropoulos A, Bresin A, Kiliaridis S. Morphometric analysis of the mandible in growing rats with different masticatory functional demands: adaptation to an upper posterior bite block. Eur J Oral Sci. 2004; 112: 259–266. doi: 10.1111/j.1600-0722.2004.00129.x 15154925

[78] Shurtliff QR. Mammalian hybrid zones: a review. Mamm Rev. 2013; 43: 1–21. doi: 10.1111/j.1365-2907.2011.00205.x

[79] Warren KA, Ritzman TB, Humphreys RA, Percival CJ., Hallgrímsson B, Ackermann RR. Craniomandibular form and body size variation of first generation mouse hybrids: a model for hominin hybridization. J Hum Evol. 2018; 116: 57–74. doi: 10.1016/j.jhevol.2017.12.002 29477182 PMC6699179

[80] Tamagnini D, Meloro C, Raia P, Maiorano L. Testing the occurrence of convergence in the cranio-mandibular shape evolution of living carnivorans. Evolution. 2021; 75: 1738–1752. doi: 10.1111/evo.14229 33844288 PMC8359831

[81] Cassini GH, Toledo N. An ecomorphological approach to craniomandibular integration in neotropical deer. J Mamm Evol. 2020; 28: 111–123. doi: 10.1007/s10914-020-09499-5

[82] Tse YT, Calede JJM. (2021). Quantifying the link between craniodental morphology and diet in the Soricidae using geometric morphometrics. Biol J Linn Soc. 2021; 133: 28–46. doi: 10.1093/biolinnean/blab005

[83] Debat V, Alibert P, David P, Paradis E, Auffray J-C. Independence between developmental stability and canalization in the skull of the house mouse. Proc Royal Soc B. 2002; 267: 423–430. doi: 10.1098/rspb.2000.1017 10737397 PMC1690549

[84] Leamy L. Morphometric studies in inbred and hybrid house mice. V. Directional and fluctuating asymmetry. Am Nat. 1984; 123: 579–593. doi: 10.1086/284225

[85] Alibert P, Renaud S, Dod B, Bonhomme F, Auffray J-C. Fluctuating asymmetry in the *Mus musculus* hybrid zone: a heterotic effect in disrupted co-adapted genomes. Proc Royal Soc B. 1994; 258: 53–59. doi: 10.1098/rspb.1994.0141 7997458

[86] Pallares LF, Turner LM, Tautz D. Craniofacial shape transition across the house mouse hybrid zone: Implications for the genetic architecture and evolution of between-species differences. Dev Genes Evol. 2016; 226: 173–186. doi: 10.1007/s00427-016-0550-7 27216933 PMC4896993

[87] Kohli BA, Jarzyna MA. Pitfalls of ignoring trait resolution when drawing conclusions about ecological processes. Glob Ecol Biogeogr. 2021; 30: 1139–1152. doi: 10.1111/geb.13275

[88] Pritchard JK, Stephens M, Donnelly P. Inference of population structure using multilocus genotype data. Genetics. 2000; 155: 945–959. doi: 10.1093/genetics/155.2.945 10835412 PMC1461096

[89] Vähä JP, Primmer CR. Efficiency of model-based Bayesian methods for detecting hybrid individuals under different hybridization scenarios and with different numbers of loci. Mol Ecol. 2006; 15: 63–72. doi: 10.1111/j.1365-294X.2005.02773.x 16367830

[90] Meers MB. Maximum bite force and prey size of *Tyrannosaurus rex* and their relationships to the inference of feeding behavior. Hist Biol. 2002; 16: 1–12. doi: 10.1080/0891296021000050755

[91] RStudio Team. RStudio: Integrated Development for R. Boston, MA: RStudio Inc. 2021.

[92] Jaslow CR. Sexual dimorphism of cranial suture complexity in wild sheep (*Ovis orientalis*). Biol J Linn Soc. 1989; 95(4): 273–284. doi: 10.1111/j.1096-3642.1989.tb02312.x

[93] White HE, Clavel J, Tucker AS, Goswami A. A comparison of metrics for quantifying cranial suture complexity. J R Soc Interface. 2020; 17: 20200476. doi: 10.1098/rsif.2020.0476 33023399 PMC7653371

[94] Rohlf FJ. tpsDig, digitize landmarks and outlines, version 2.32. Department of Ecology and Evolution, State University of New York at Stony Brook. 2005.

[95] Adams DC, Otárola-Castillo E. (2013). Geomorph: An R package for the collection and analysis of geometric morphometric shape data. Methods Ecol Evol. 2013; 4: 393–399. doi: 10.1111/2041-210X.12035

[96] Perez SI, Bernal V, Gonzalez PN. Differences between sliding semi-landmark methods in geometric morphometrics, with an application to human craniofacial and dental variation. J Anat. 2006; 208: 769–784. doi: 10.1111/j.1469-7580.2006.00576.x 16761977 PMC2100233

[97] Bookstein FL. Morphometric tools for landmark data: geometry and biology. Cambridge UK: Cambridge University Press. 1997. doi: 10.1017/CBO9780511573064

[98] Zelditch ML, Swiderski DL, Sheets HD. Geometric morphometrics for biologists: a primer. New York: Academic Press. 2012. doi: 10.1016/B978-0-12-778460-1.X5000-5

[99] Biostats McGarigal K. 2015.

[100] Swanson HK, Lysy M, Power M, Stasko AD, Johnson JD, Reist JD. A new probabilistic method for quantifying n-dimensional ecological niches and niche overlap. Ecology. 2015; 96: 318–324. doi: 10.1890/14-0235.1 26240852

[101] Hedrick BP. Inter- and intraspecific variation in *Artibeus* species complex demonstrates size and shape partitioning among species. Peer J. 2021; 9:e11777. doi: 10.7717/peerj.11777 34306832 PMC8280882

[102] Machado FA, Zahn TMG, Marroig G. Evolution of morphological integration in the skull of Carnivora (Mammalia): Changes in Canidae lead to increased evolutionary potential of facial traits. Evolution. 2018; 72: 1399–1419. doi: 10.1111/evo.13495 29803199

