# Supplementary material for: Craniodental divergence associated with bite force between hybridizing pine squirrels (Tamiasciurus)
Source: PLoS One. 2023 Apr 6;18(4):e0284094. doi: 10.1371/journal.pone.0284094 (PMC10079020; doi:10.1371/journal.pone.0284094)
Supplement: S1 Fig — Data shown are a posterior histogram of 3000 iterations of overlap metric calculations, whereby the x-axis of each pairwise comparison is the range of overlap probabilities in percentage and the y-axis is frequency of that probability having been observed in calculation. Comparisons are for morphospace overlap between the squirrel group in the grid row on the squirrel group in the grid column. Mean overlap probability is shown as a solid black line and 95% confidence intervals are shown as dashed lines for all pairs. (DOCX) [file pone.0284094.s001.docx]

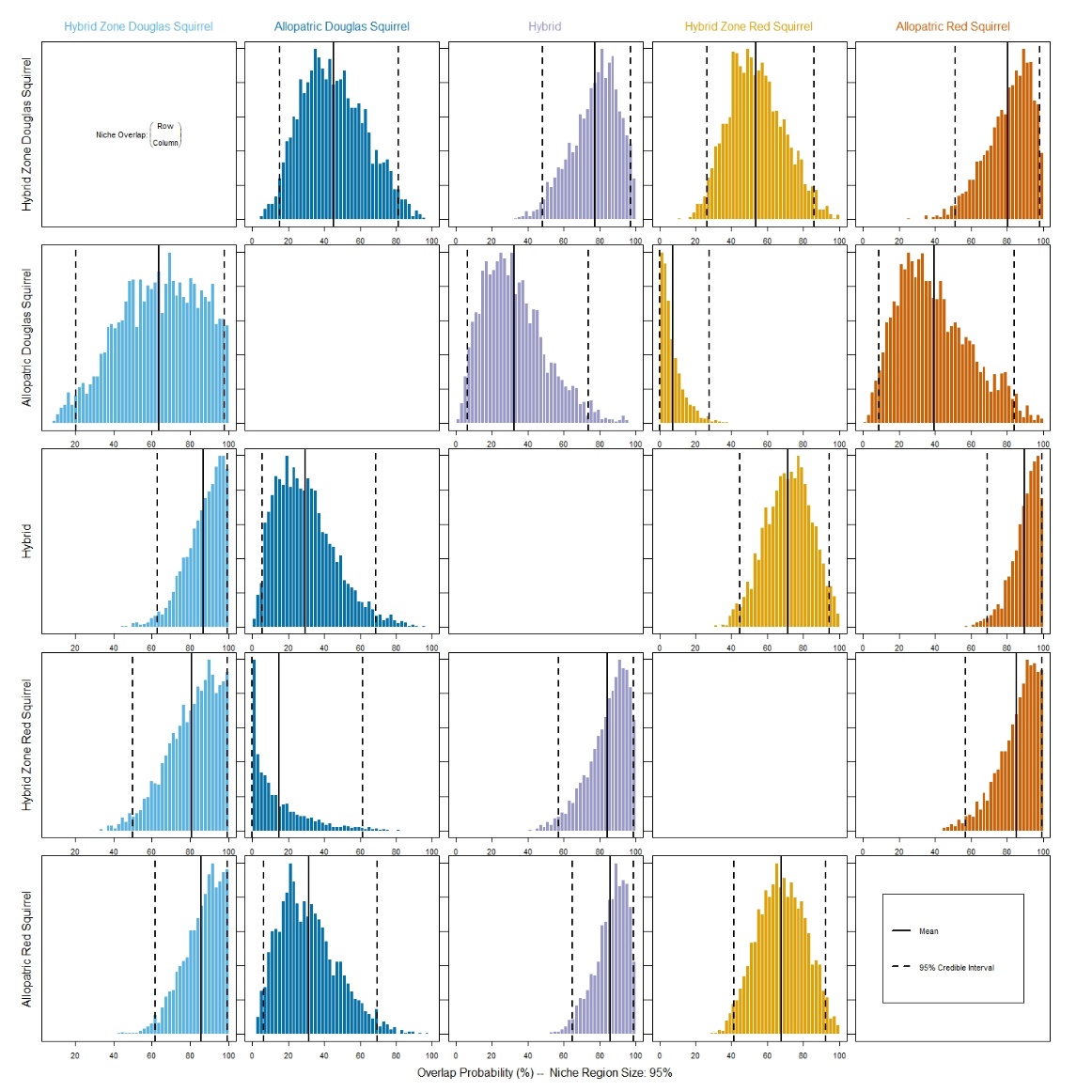


**Fig S1.** Analysis of overlap probability of mandible geometric morphometric principal components 1 and 2 representing shape and log10-transformed centroid size representing size. Data shown are a posterior histogram of 3000 iterations of overlap metric calculations, whereby the x-axis of each pairwise comparison is the range of overlap probabilities in percentage and the y-axis is frequency of that probability having been observed in calculation. Comparisons are for morphospace overlap between the squirrel group in the grid row on the squirrel group in the grid column. Mean overlap probability is shown as a solid black line and 95% confidence intervals are shown as dashed lines for all pairs.
