# Supplementary material for: Craniodental divergence associated with bite force between hybridizing pine squirrels (Tamiasciurus)
Source: PLoS One. 2023 Apr 6;18(4):e0284094. doi: 10.1371/journal.pone.0284094 (PMC10079020; doi:10.1371/journal.pone.0284094)
Supplement: S2 Fig — (A) Douglas squirrel cranium from specimen UWBM 20809. (B) Douglas squirrel left mandible from specimen UWBM 20809, image is flipped. (C) Hybrid squirrel cranium from specimen UWBM 21078. (D) Hybrid squirrel right mandible from specimen UWBM 21078. (E) Red squirrel cranium from specimen UWBM 21034. (F) Red squirrel left mandible from specimen UWBM 21034, image is flipped. (DOCX) [file pone.0284094.s002.docx]

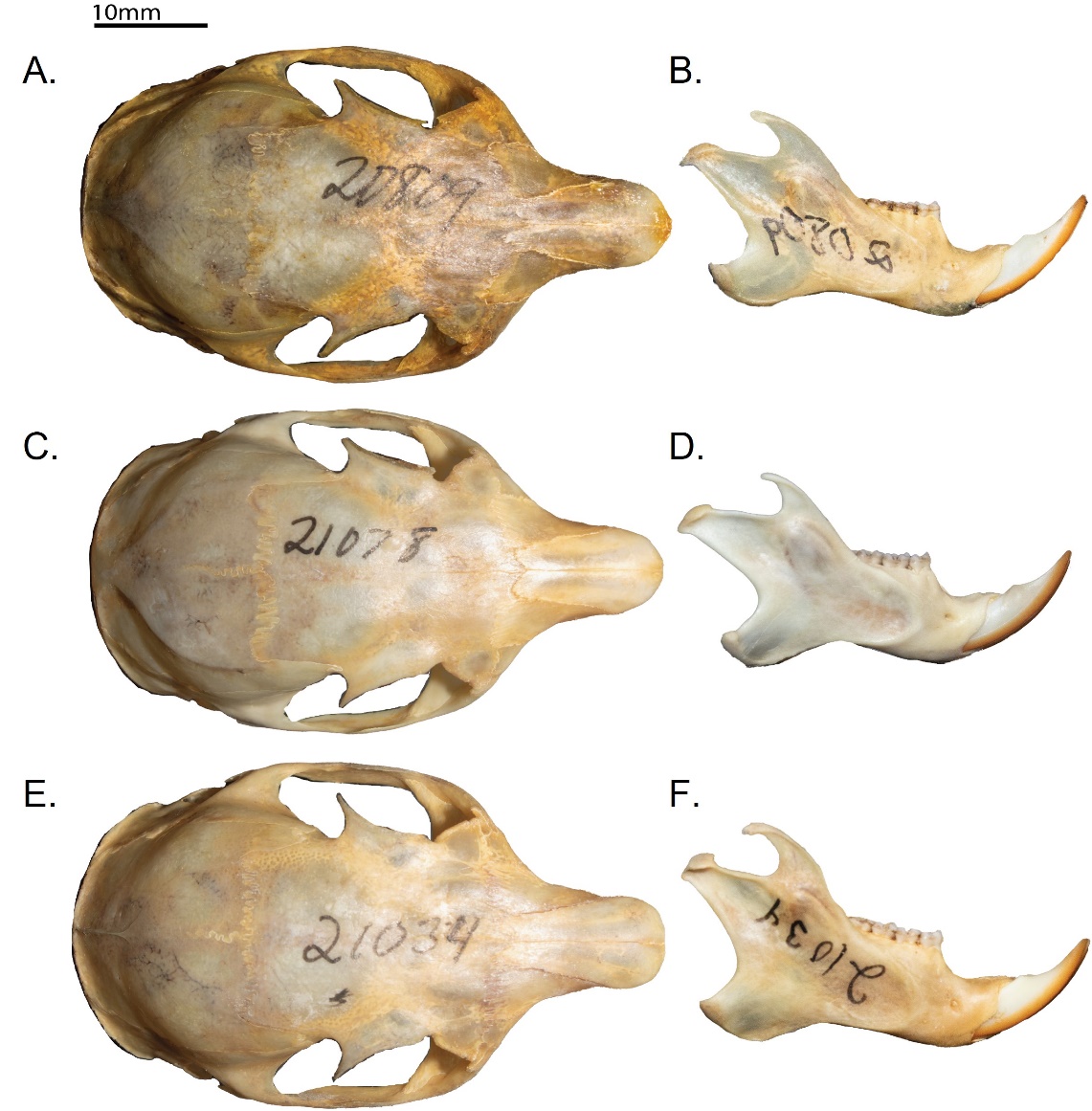


**Fig S2.** Representative examples for hybrid zone specimens. **A.** Douglas squirrel cranium from specimen UWBM 20809. **B.** Douglas squirrel left mandible from specimen UWBM 20809, image is flipped. **C.** Hybrid squirrel cranium from specimen UWBM 21078. **D.** Hybrid squirrel right mandible from specimen UWBM 21078. **E.** Red squirrel cranium from specimen UWBM 21034. **F.** Red squirrel left mandible from specimen UWBM 21034, image is flipped.
