# Supplementary material for: Craniodental divergence associated with bite force between hybridizing pine squirrels (Tamiasciurus)
Source: PLoS One. 2023 Apr 6;18(4):e0284094. doi: 10.1371/journal.pone.0284094 (PMC10079020; doi:10.1371/journal.pone.0284094)
Supplement: S2 Table — (DOCX) [file pone.0284094.s004.docx]

| **Table S2. Homologous landmark descriptions** | |
| --- | --- |
| **ID** | **Location** |
| 1 | Antero-dorsal border of the incisor |
| 2 | Invagination of the diastema |
| 3 | Anterior of the molar tooth-row |
| 4 | Boundary between M1 and M2 |
| 5 | Molar tooth-row and coronoid process intersection |
| 6 | Tip of the coronoid process |
| 7 | Most concave point between coronoid and condylar processes |
| 8 | Anterior edge of mandibular condyle |
| 9 | Posterior edge of mandibular condyle |
| 10 | Tip of the angular process |
| 11 | Ventral point of the angular process |
| 12 | Most concave point of horizontal ramus |
| 13 | Posterior point of mandibular symphysis |
| 14 | Antero-ventral border of incisor |
| 15 | Anterior edge of masseteric ridge |
