# Supplementary material for: Craniodental divergence associated with bite force between hybridizing pine squirrels (Tamiasciurus)
Source: PLoS One. 2023 Apr 6;18(4):e0284094. doi: 10.1371/journal.pone.0284094 (PMC10079020; doi:10.1371/journal.pone.0284094)
Supplement: S3 Table — (DOCX) [file pone.0284094.s005.docx]

| **Table S3. Semilandmark assignment and descriptions** | | | |
| --- | --- | --- | --- |
| **Curve** | **Curve location** | **Region** | **Number of semilandmarks** |
| 1 | Between landmarks 1 and 2 | Antero-dorsal edge of the diastema | 4 |
| 2 | Between landmarks 5 and 6 | Dorsal edge of the coronoid process | 11 |
| 3 | Between landmarks 6 and 7 | Curve between tip of coronoid process to the most concave point of the incisura mandibulare | 6 |
| 4 | Between landmarks 7 and 8 | Continuation of incisura mandibulare curve to the anterior edge of the mandibular condyle | 6 |
| 5 | Between landmarks 8 and 9 | Posterior edge of mandibular condyle | 4 |
| 6 | Between landmarks 9 and 10 | Curve of horizontal ramus | 9 |
| 7 | Between landmarks 11 and 12 | Ventral border of angular process | 2 |
| 8 | Between landmarks 12 and 13 | Ventral border of molar zone | 3 |
