# Supplementary material for: Craniodental divergence associated with bite force between hybridizing pine squirrels (Tamiasciurus)
Source: PLoS One. 2023 Apr 6;18(4):e0284094. doi: 10.1371/journal.pone.0284094 (PMC10079020; doi:10.1371/journal.pone.0284094)
Supplement: Attachment — Submitted filename: PONE-D-22-29005_review.pdf [file pone.0284094.s006.pdf]

PLOS ONE

Craniodental divergence associated with bite force between hybridizing pine squirrels (Tamiasciurus)

--Manuscript Draft--

**Manuscript Number:**	PONE-D-22-29005
**Article Type:**	Research Article
**Full Title:**	Craniodental divergence associated with bite force between hybridizing pine squirrels (Tamiasciurus)
**Short Title:**	Craniodental divergence in hybridizing squirrels
**Corresponding Author:**	Dylan Matthew Poorboy, M.Sc.<br>The Ohio State University<br>Columbus, OH UNITED STATES
**Keywords:**	Bite force, ecomorphology, geometric morphometrics, hybridization, Sciuridae, suture complexity
**Abstract:**	Bite force can be a limiting factor in foraging and can have significant consequences on the competitive ability and lifetime fitness in mammals. Tamiasciurus squirrels feed primarily on conifer seeds and have a strong bite force for mechanically extracting seeds from conifer cones with their mouths. In the North Cascades region, Douglas squirrels (Tamiasciurus douglasii) and red squirrels (T. hudsonicus) occupy ecologically different forests with different hardnesses in conifer cones. These squirrel species meet in a narrow hybrid zone where these forests meet near the crest of the North Cascades. We examined interspecific divergence in dietary ecomorphology in allopatry, in sympatry within the hybrid zone, and between hybrids and each parental species. We focused on three craniodental traits, including the incisor-strength index as a proxy measure for bite force, cranial-suture complexity, and mandible shape. We find these sister squirrel species are different in bite force and suture complexity in both allopatry and sympatry and that mandible shape changes with the expected hardness of accessed food items, but not directly between species. Furthermore, we find that hybrids display morphologies that overlap with sympatric red squirrels but not with sympatric Douglas squirrels. This work shows how important ecological processes at shallow evolutionary timescales can impact the divergence of morphological traits in taxa with extreme conservation of craniomandibular shape.
**Order of Authors:**	Dylan Matthew Poorboy, M.Sc.
	Jonathan J.-M. Calède, Ph.D.
	Andreas S. Chavez, Ph.D.
**Additional Information:**	
**Question**	**Response**
**Financial Disclosure**<br><br>Enter a financial disclosure statement that describes the sources of funding for the work included in this submission. Review the submission guidelines for detailed requirements. View published research articles from *PLOS ONE* for specific examples.<br><br>This statement is required for submission and **will appear in the published article** if the submission is accepted. Please make sure it is accurate.	The authors received no specific funding for this work.

Powered by Editorial Manager® and ProduXion Manager® from Aries Systems Corporation

**Unfunded studies**<br>Enter: *The author(s) received no specific funding for this work.*<br><br>**Funded studies**<br>Enter a statement with the following details:<br>• Initials of the authors who received each award<br>• Grant numbers awarded to each author<br>• The full name of each funder<br>• URL of each funder website<br>• Did the sponsors or funders play any role in the study design, data collection and analysis, decision to publish, or preparation of the manuscript?<br>• **NO** - Include this sentence at the end of your statement: *The funders had no role in study design, data collection and analysis, decision to publish, or preparation of the manuscript.*<br>• **YES** - Specify the role(s) played.<br><br>* typeset	
**Competing Interests**<br><br>Use the instructions below to enter a competing interest statement for this submission. On behalf of all authors, disclose any competing interests that could be perceived to bias this work—acknowledging all financial support and any other relevant financial or non-financial competing interests.<br><br>This statement is required for submission and **will appear in the published article** if the submission is accepted. Please make sure it is accurate and that any funding sources listed in your Funding Information later in the submission form are also declared in your Financial Disclosure statement.<br><br>View published research articles from *PLOS ONE* for specific examples.	The authors have declared that no competing interests exist.

**NO authors have competing interests**<br><br>Enter: *The authors have declared that no competing interests exist.*<br><br>**Authors with competing interests**<br><br>Enter competing interest details beginning with this statement:<br><br>*I have read the journal's policy and the authors of this manuscript have the following competing interests: [insert competing interests here]*<br><br>* typeset	
**Ethics Statement**<br><br>Enter an ethics statement for this submission. This statement is required if the study involved:<br><br>• Human participants<br>• Human specimens or tissue<br>• Vertebrate animals or cephalopods<br>• Vertebrate embryos or tissues<br>• Field research<br><br>Write "N/A" if the submission does not require an ethics statement.<br><br>General guidance is provided below. Consult the submission guidelines for detailed instructions. **Make sure that all information entered here is included in the Methods section of the manuscript.**	All specimens used in this study were acquired from museum collections.

**Format for specific study types**<br><br>**Human Subject Research (involving human participants and/or tissue)**<br>• Give the name of the institutional review board or ethics committee that approved the study<br>• Include the approval number and/or a statement indicating approval of this research<br>• Indicate the form of consent obtained (written/oral) or the reason that consent was not obtained (e.g. the data were analyzed anonymously)<br><br>**Animal Research (involving vertebrate animals, embryos or tissues)**<br>• Provide the name of the Institutional Animal Care and Use Committee (IACUC) or other relevant ethics board that reviewed the study protocol, and indicate whether they approved this research or granted a formal waiver of ethical approval<br>• Include an approval number if one was obtained<br>• If the study involved *non-human primates*, add *additional details* about animal welfare and steps taken to ameliorate suffering<br>• If anesthesia, euthanasia, or any kind of animal sacrifice is part of the study, include briefly which substances and/or methods were applied<br><br>**Field Research**<br><br>Include the following details if this study involves the collection of plant, animal, or other materials from a natural setting:<br>• Field permit number<br>• Name of the institution or relevant body that granted permission	
**Data Availability**<br><br>Authors are required to make all data underlying the findings described fully available, without restriction, and from the time of publication. PLOS allows rare exceptions to address legal and ethical concerns. See the PLOS Data Policy and FAQ for detailed information.	Yes - all data are fully available without restriction

Powered by Editorial Manager® and ProduXion Manager® from Aries Systems Corporation

A Data Availability Statement describing where the data can be found is required at submission. Your answers to this question constitute the Data Availability Statement and **will be published in the article**, if accepted.<br><br>**Important:** Stating 'data available on request from the author' is not sufficient. If your data are only available upon request, select 'No' for the first question and explain your exceptional situation in the text box.<br><br>Do the authors confirm that all data underlying the findings described in their manuscript are fully available without restriction?	
**Describe where the data may be found in full sentences. If you are copying our sample text, replace any instances of XXX with the appropriate details.**<br><br>• If the data are **held or will be held in a public repository**, include URLs, accession numbers or DOIs. If this information will only be available after acceptance, indicate this by ticking the box below. For example: *All XXX files are available from the XXX database (accession number(s) XXX, XXX.).*<br>• If the data are all contained **within the manuscript and/or Supporting Information files**, enter the following: *All relevant data are within the manuscript and its Supporting Information files.*<br>• If neither of these applies but you are able to provide **details of access elsewhere**, with or without limitations, please do so. For example:<br><br>*Data cannot be shared publicly because of [XXX]. Data are available from the XXX Institutional Data Access / Ethics Committee (contact via XXX) for researchers who meet the criteria for access to confidential data.*<br><br>*The data underlying the results presented in the study are available from (include the name of the third party*	All data files can be and will be made accessible after acceptance for publication

and contact information or URL). • This text is appropriate if the data are owned by a third party and authors do not have permission to share the data. <br>* typeset	
Additional data availability information:	Tick here if the URLs/accession numbers/DOIs will be available only after acceptance of the manuscript for publication so that we can ensure their inclusion before publication.

Manuscript
Click here to access/download;Manuscript;Poorboy_etal_HybridZoneManuscri

Craniodental divergence associated with bite force between hybridizing pine squirrels (*Tamiasciurus*)

Dylan M. Poorboy[1], Jonathan J.-M. Calède[1,2], Andreas S. Chavez[1,3]

Short title: Craniodental divergence in hybridizing squirrels

[1]Department of Evolution, Ecology and Organismal Biology, The Ohio State University, Columbus, OH, United States
[2]The Ohio State University at Marion, 1459 Mount Vernon Avenue, Marion, OH 43302, USA
[3]Translational Data Analytics Institute, The Ohio State University, Columbus, OH, United States

Corresponding Author: Dylan M. Poorboy (poorboy.1@buckeyemail.osu.edu)

Abstract

Bite force can be a limiting factor in foraging and can have significant consequences on the competitive ability and lifetime fitness in mammals. *Tamiasciurus* squirrels feed primarily on conifer seeds and have a strong bite force for mechanically extracting seeds from conifer cones with their mouths. In the North Cascades region, Douglas squirrels (*Tamiasciurus douglasii*) and red squirrels (*T. hudsonicus*) occupy ecologically different forests with different hardnesses in conifer cones. These squirrel species meet in a narrow hybrid zone where these forests meet near the crest of the North Cascades. We examined interspecific divergence in dietary ecomorphology in allopatry, in sympatry within the hybrid zone, and between hybrids and each parental species. We focused on three craniodental traits, including the incisor-strength index as a proxy measure for bite force, cranial-suture complexity, and mandible shape. We find these sister squirrel species are different in bite force and suture complexity in both allopatry and sympatry and that mandible shape changes with the expected hardness of accessed food items, but not directly between species. Furthermore, we find that hybrids display morphologies that overlap with sympatric red squirrels but not with sympatric Douglas squirrels. This work shows how important ecological processes at shallow evolutionary timescales can impact the divergence of morphological traits in taxa with extreme conservation of craniomandibular shape.

Introduction

The ecological processes that generate morphological divergence can be challenging to discern in comparative studies focused on species across deep taxonomic timescales. Ecomorphology

can be the product of several, at times opposing, selective processes that may not persist through the entire evolutionary history of deep lineages. At shallow timescales, ~~ecological~~ processes, including divergent ecological selection, hybridization, and interspecific competition, can be important initiators of ecomorphological diversity [1-3]. Hybrid zones representing secondary contact and hybridization between ecologically divergent species are practical arenas to investigate how these three ~~ecological~~ processes impact variation on ecomorphology at shallow timescales. In hybrid zones that span ecological transitions, differences in phenotypic variation between species is subject to the homogenizing effects of gene flow and its counterposing force, divergent selection [4-6]. Furthermore, interspecific competition within hybrid zones can potentially drive the evolution of character [7-9] or, in contrast, phenotypic convergence [10-12].

Hybridization, in the absence of evolutionary forces, can have significant consequences on phenotypic variation. Hybridization leads to novel epistatic interactions between differentiated genomes, which may allow for a change in morphospace occupation due to a relaxation of trait covariation [13-14]. Thus, hybridization can immediately lead to several phenotypic possibilities, including (1) hybrid phenotypes that are intermediate between the parental phenotypes [15-16]; (2) hybrid phenotypes that resemble one of the parental phenotypes, reflecting genetic dominance [17-19]; or (3) transgressive hybrid phenotypes that are more extreme than either parental phenotype [20-24]. The consequences of evolutionary forces on hybrid phenotypes are also varied and range from strong selection against hybrid morphologies [25] to selection that favors novel hybrid morphologies [2,3].

Ecomorphological traits associated with foraging are routinely under strong natural selection for functional performance and illustrate the combination of both precise local adaptation and extreme evolutionary diversification [26]. In mammals, several craniodental traits are associated with generating and sustaining maximal bite force. First, the incisor strength index is a reliable proxy for maximal bite force in rodents [27]. This index is based on a rectangular cross-section of the incisor and its ability to resist a bending force, which allows it to be a valuable estimator of bite force where *in vivo* measurements are challenging to acquire or consistently replicate due to variable behavior [27]. Next, the suture lines of the cranium reveal the counterforce stresses that the cranium receives from biting on food items [28-32]. Consequently, suture complexity is positively associated with greater shock absorption and redistribution ability resulting from stronger bite force [33,34]. Finally, mandible shape may influence muscle attachment and mechanical levers, with the expectation that more durophagous animals will have larger or more robust processes and higher mechanical lever advantages [35-38].

The hybrid zone between ecologically divergent Douglas squirrels (*T. douglasii*) and red squirrels (*T. hudsonicus*) represents an exemplary opportunity to study the consequences of ecologically divergent selection, interspecific competition, and hybridization on craniodental traits associated with bite force. This parapatric pair of squirrel species diverged less than a million years ago and are hybridizing in a narrow secondary contact zone near the crest of the North Cascades region in northern Washington and southern British Columbia (Fig 1; [39-41]). In this region, Douglas squirrels occupy wet maritime forests on the west side of the Cascade Range, and red squirrels occupy dry continental forests on the east side. Both squirrel species

feed primarily on conifer seeds by mechanically removing scales off conifer cones with their mouth.

In the North Cascades hybrid zone, ecological divergent selection may be an important driver of divergence in craniodental traits associated with bite force because of differences in the hardness of conifer cones found in the respective forests of Douglas squirrels and red squirrels. Seeds from lodgepole pine are an important food for red squirrels in dry continental forests. These seeds are encased in hard, serotinous (closed) cones. In contrast, all conifer species in the wet maritime forests in the range of Douglas squirrels produce relatively softer and more opened cones [42-44]. One of the most striking craniodental differences between these squirrel species due to their different biomechanical challenges of procuring conifer seeds is the prominent sagittal crest found in red squirrels and the shorter and medio-laterally expanded sagittal “plateau” found in Douglas squirrels [40,45]. Comparisons of other craniodental traits associated with bite force between these squirrel species living in different forests will help shed light on whether divergent selection has generated greater divergence in other parts of the skull than previously understood.

Fig 1. Map of the North Cascades region showing range of Douglas squirrels (blue), red squirrels (red), and the hybrid zone where ranges overlap (purple). Some specimens within the hybrid zone geographically overlap, see Supplement 1 for complete specimen details.

Both squirrel species and their hybrids are syntopic in the hybrid zone, and thus interspecific competition may have important consequences on access to food resources and,

ultimately, the evolution of craniodental morphologies associated with foraging. *Tamiasciurus* squirrels vigorously defend territories that are centered around their cache of conifer cones [42,46]. One potential outcome of intense interspecific competition is the evolution of ecological character displacement in response to selection that lessens resource competition between interacting species [7]. However, character displacement and competitive exclusion in hybrid-zone systems may be prevented by the co-existence and viability of hybrids with intermediate phenotypes [47]. Comparisons of ecomorphologies between hybrid zone populations and allopatric populations for each parental squirrel species can begin to uncover whether interspecific competition is an important factor driving morphological change.

Hybrid squirrels establish territories amongst both parental species within the same forest and thus have similar food habits [39, 46]. The creative effects of hybridization may allow hybrid squirrels to have morphologies outside the range of either species and potentially allow them to be either more or less effective at exploiting food resources than either parental species. Comparisons of ecomorphologies between hybrid squirrels and each parental species can be helpful ~~in beginning~~ to understand whether hybrids forage similarly or not.

Here, we conduct a comparative study of bite force strength, suture complexity, and mandible shape to determine whether divergence in these functional traits is consistent with ecological divergent selection, interspecific competition, and hybridization. Specifically, we investigate the following three hypotheses on the patterns of ecomorphological differences associated with bite force: (1) differences between allopatric Douglas squirrels and red squirrels are consistent with divergent selection due to major differences in cone hardness; (2) differences between sympatric Douglas squirrels and red squirrels are consistent with character

displacement resulting from intense interspecific competition for shared food resources; and (3) differences between hybrids and parental species are consistent with hybridization generating morphologies outside of the range of either parental species.

Results

Bite Force

BFQ differs significantly across squirrel groups (Fig 2; $p<0.0001$, $F_{4,55} = 8.86$). Pairwise comparisons reveal that the BFQ is lower for Douglas squirrels than for red squirrels, both in allopatry ($p < 0.0001$) and in sympatry within the hybrid zone ($p = 0.014$). The hybrid zone red squirrel population has a lower BFQ than the allopatric red squirrel population ($p = 0.014$). Similarly, the hybrid zone Douglas squirrel population has a lower BFQ than the allopatric Douglas squirrel population ($p = 0.036$). The BFQ of hybrids is not significantly different from that of sympatric hybrid-zone red squirrels ($p=0.94$), but it is significantly greater than that of sympatric hybrid-zone Douglas squirrels ($p=0.016$).

Fig 2. Boxplot showing variation in bite force quotient (BFQ) for each group of squirrels. Values on comparison bars represent significant p-values from pairwise comparisons. Outliers are indicated by black dots. Abbreviations: Allopatric Douglas squirrel, Allo Doug; Hybrid Zone Douglas squirrel, HZ Doug; Hybrid Zone red squirrel, HZ Red; and allopatric red squirrel, Allo Red.

Suture Morphology

We find significant differences in suture complexity (LR) among squirrel groups for the premaxillofrontal (Fig 3B; $p < 0.001$, $F_{4,54} = 6.81$), maxillofrontal (Fig 3D; $p = 0.0027$, $F_{4,53} = 4.64$), and sagittal sutures (Fig 3F; $p < 0.0001$, $F_{4,50} = 10.20$), but no differences for either the nasofrontal (Fig 3C; $p = 0.182$, $F_{4, 54} = 1.62$) or coronal sutures (Fig 3E; $p = 0.223$, $F_{4, 53} = 1.48$). Our post-hoc pairwise comparisons demonstrate significant differences between allopatric red squirrels and allopatric Douglas squirrels for the premaxillofrontal ($p = 0.0041$), maxillofrontal ($p = 0.0021$), and sagittal ($p = 0.0002$) sutures. We also find significant pairwise differences between the hybrid-zone red squirrels and the hybrid-zone Douglas squirrels for the premaxillofrontal ($p = 0.0013$), maxillofrontal ($p = 0.037$), and sagittal ($p = 0.01$) sutures. Hybrids are only significantly different from the hybrid-zone Douglas squirrels in their premaxillofrontal ($p = 0.0047$) and sagittal sutures ($p = 0.028$), but not in their maxillofrontal suture ($p = 0.36$). Hybrids are not significantly different from hybrid-zone red squirrels in any of their sutures

The first two axes of the PCA of all five sutures, PC1 and PC2, explain 65.95% of the variation in suture complexity (Fig 3A; PC1: 45.95%; PC2: 20.00%). PC1 is primarily driven by the complexity of the premaxillofrontal, maxillofrontal, and sagittal sutures with negative PC scores representing greater complexity for these three sutures. PC2 is primarily determined by variation in coronal and nasofrontal sutures; with increased complexity in the nasofrontal suture associated with positive PC scores, whereas increased complexity in coronal sutures is associated with negative PC scores. An ANOVA reveals a significant difference in PC1 scores among squirrel groups ($p < 0.0001$, $F_{4,48} = 10.05$), but no significant difference is present for PC2 among groups ($p = 0.482$, $F_{4,48} = 0.51$). Post-hoc tests reveal a significant difference in PC1 scores between Douglas squirrels and red squirrels in allopatry ($p=0.0002$), as well as in

sympatry (p=0.013), however, no significant differences in PC1 scores between allopatric Douglas squirrel and hybrid-zone Douglas squirrels (p = 0.73) or between allopatric red squirrels and hybrid-zone red squirrels (p=0.84). Hybrids differ significantly in PC1 scores from hybrid-zone Douglas squirrels (p=0.0379), but not from hybrid-zone red squirrels (p=0.386).

Fig 3. Suture complexity comparisons between Tamiasciurus groups. Values on comparison bars represent significant p-values from pairwise comparisons. Outliers are indicated by black dots. Abbreviations are provided in Fig 2. **(A).** Principal component analysis for all five sutures. **(B).** ANCOVA for the premaxillofrontal suture. **(C).** ANCOVA for the nasofrontal suture. **(D).** ANCOVA for the maxillofrontal suture. **(E).** ANCOVA for the coronal suture. **(F).** ANCOVA for the sagittal suture.

Hybrid admixture regressions

A minimum admixture score of 0.18 and maximum admixture score of 0.83 were calculated for the hybrids studied, with a mean score of 0.5353 (± 0.0749 95% CI) for the 19 hybrid individuals. Regressions of admixture proportion for hybrids and their measured dietary ecomorphological traits were tested for genetic correlations. Admixture proportions (Q) were not significantly correlated with BFQ (R^2 = 0.01, p = 0.771) or any of the five LR measurements (nasofrontal: R^2 = 0.01, p = 0.627; premaxillofrontal: R^2 = 0.001, p = 0.879; maxillofrontal: R^2 = 0.02, p = 0.558; coronal: R^2 = 0.04, p = 0.464; sagittal: R^2 = 0.03, p = 0.468).

Geometric Morphometrics

Monte Carlo randomization supported PC1 and PC2 as significant axes for the morphospace PCA, thus, only these axes were retained for subsequent analyses. PC1 and PC2 explain 45.97% of the variation in dentary morphology among the squirrels studied (Fig 4; PC1: 35.57%; PC2: 10.40%). Specimens with more positive PC1 values have an anteroposteriorly shortened dentary, a ~~dorsoventrally~~ shortened condylar process, and ~~a dorsoventrally heightened~~ angular process. Positive PC2 values are associated with an anteroposteriorly longer and more dorsoventrally curved and pointed coronoid process but an anteroposteriorly reduced angular process.

Fig 4. Principal component analysis of mandible shape in lateral view. Deformation grids for principal component axes accompany their respective axis. Abbreviations are provided in Fig 2.

We analyzed the possible effects of centroid size and sex on mandible shape to ascertain the roles of allometric scaling and sexual dimorphism. The Procrustes ANOVA showed that mandible shape is significantly correlated with log10-transformed centroid size (Table 1; $p = 0.001$, $F_{1,46} = 5.19$) and the interaction between group and centroid size ($p=0.007$, $F_{4,46} = 2.38$); but mandible shape was not significantly affected by sex ($p = 0.264$, $F_{1,46} = 0.61$) or interactions between sex and size ($p = 0.166$, $F_{1,46} = 0.99$). The ANOVA of centroid size demonstrated a significant difference in size among the different squirrel groups (Fig 5; $p < 0.001$, $F_{4,53} = 22.94$). Post-hoc comparisons indicate significant differences in centroid size between allopatric red squirrels and allopatric Douglas squirrels ($p < 0.0001$), as well as between hybrid-zone red squirrels and hybrid-zone Douglas squirrels ($p = 0.0011$). In addition, hybrid-zone Douglas

squirrels are significantly larger than allopatric Douglas squirrels (p<0.0001). There are no significant differences between hybrid-zone red squirrels and allopatric red squirrels (p = 0.498). Hybrids are significantly larger than hybrid-zone Douglas squirrels (p =0.022) but are not different from hybrid-zone red squirrels (p = 0.13). The Procrustes ANOVA indicates a significant effect of group on mandible shape (p = 0.001, $F_{4,46}$ = 6.41); however, there were no significant pairwise comparisons for Procrustes data after correction for multiple testing.

Fig 5. Variation in log10-transformed mandible centroid size across squirrel groups. Values on comparison bars represent significant p-values from pairwise comparisons. Outliers are indicated by black dots. Abbreviations are provided in Fig 2.

Table 1. Procrustes ANOVA of lateral view mandible shape data. Relationships assessed were for squirrel group, sex, log transformed centroid size, and the interactions size by group and size by sex. Significant p-values are shown in bold.

	Df	SS	MS	Rsq	F	Z	Pr(>F)
Group	4	0.002426	0.000606	0.38768	13.3115	6.4123	**0.001**
Sex	1	5.74E-05	5.74E-05	0.00918	1.2603	0.6071	0.264
Size	1	0.001139	0.001139	0.18198	24.9946	5.1933	**0.001**
Group:Size	4	0.000455	0.000114	0.07271	2.4967	2.3804	**0.007**
Sex:Size	1	8.47E-05	8.47E-05	0.01353	1.8587	0.9875	0.166
Residuals	46	0.002095	4.56E-05	0.33492			
Total	57	0.006257					

The analysis of morphospace overlap using nicheROVER reveals varying degrees of overlap in dentary shape and size among squirrel groups (Fig 6; Fig S1). Allopatric Douglas squirrels show a high degree of overlap with hybrid-zone Douglas squirrels but low overlap with allopatric red squirrels, hybrid-zone red squirrels, and hybrids. Hybrid-zone Douglas squirrels show a high degree of overlap with hybrids and a greater probability of overlap with red

squirrel populations than with Douglas squirrels in the parental range. Hybrids have a strong probability of overlapping with all other squirrel groups except for allopatric Douglas squirrels. Finally, both allopatric red squirrels and hybrid-zone red squirrels have a high overlap probability with other squirrel groups, except allopatric Douglas squirrels.

Fig 6. Analysis of overlap probability of mandible geometric morphometric principal components 1 and 2 representing shape and log10-transformed centroid size representing size. Data shown are a posterior histogram of 3000 iterations of overlap metric calculations, whereby the x-axis of each pairwise comparison is the range of overlap probabilities in percentage, and the y-axis is the frequency of that probability having been observed in the calculation. Comparisons are for morphospace overlap between the squirrel group labeled to the left of each graph on the squirrel group labeled above each graph. Mean overlap probability is shown as a solid black line, and 95% confidence intervals are shown as dashed lines for all pairs. The plots shown here are for the comparisons indicated for each set of plots, additional comparisons are shown in Fig S1.

Discussion

The dietary ecomorphology of an organism is phenotypically complex, involving multiple integrated morphological traits that enable interactions with the environment [48-52]. This is especially relevant for mammal skulls, where food acquisition and processing are determined mainly by muscle attachments, muscle masses, and mechanical levers that control bite force and, therefore, the food items that can be accessed [53-55]. In this study, we investigated the

evolution of three morphological traits associated with feeding, including incisor shape as a measure of bite force, cranial suture complexity, and mandible shape. We found a consistent pattern across all morphological traits that red squirrels have traits for stronger bite force than Douglas squirrels in both allopatry and sympatry, as well as a tendency for the hybrids to more closely resemble red squirrels than Douglas squirrels in morphology.

Inter-parental ecomorphological differences in allopatry

We find support for our hypothesis that ecomorphological divergence between allopatric red squirrels and Douglas squirrels is consistent with expectations under strong ecological divergent selection; this is true for all morphological traits studied. Red squirrels possess a stronger bite force (as measured by their incisor strength index), as well as greater suture complexity than Douglas squirrels. In these allopatric populations, it has been previously documented that red squirrels possess a prominent sagittal crest, associated with an expectedly larger temporalis muscle for stronger gnawing of hard conifer cones, whereas Douglas squirrels display a lower sagittal "plateau" [40,45]. The sagittal crest forms along the sagittal suture, thus, our findings of greater sagittal suture complexity in red squirrels are consistent with their stronger temporalis muscles exerting greater forces to this suture. Furthermore, we also find greater complexity in the premaxillofrontal and maxillofrontal sutures in red squirrels. Greater cranial suture complexity is expected to be important in alleviating the forces associated with a strong biting action [28-32]. These specific sutures are associated with tooth-bearing bones and are particularly expected to be more complex where masticatory strains are greater [56]. The stronger biting ability of red squirrels is also apparent

in their mandible shape. Allopatric red squirrels have a shorter mandibular corpus, a ~~dorsoventrally~~ shorter condylar process, and a ~~dorsoventrally~~ longer angular process than allopatric Douglas squirrels. This morphological shape in red squirrels allows them a relatively greater mechanical advantage. This pattern is consistent with the general pattern defining nut-eating squirrels in which they have been characterized as having a robust mandibular corpus, high ramus, and well-developed processes [35]. Thus, the foundation of morphological divergence between allopatric red squirrels and Douglas squirrels appears to be occurring in the components of the mandible most directly associated with a durophagous diet. Our findings of ecomorphological divergence in the craniomandibular shape between *Tamiasciurus* species are interesting in the context of tree squirrels, which have skulls that are generally considered to be extreme examples of morphological conservation [35,38,57-60].

The morphological differences in red squirrels that enable stronger bite force is consistent with the greater hardness of their primary food item. Seeds from lodgepole pine are a primary food item for red squirrels east of the Cascade Range. The dry climate in this region allows for high-intensity fires that threaten forest establishment. Lodgepole pine has evolved the ability to produce serotinous (closed) cones that only open during forest fires to protect their seeds during fires. Serotinous cones are harder in texture and have reduced seed numbers than non-serotinous cones [61-63]. However, the hard lodgepole pine cone creates an important selective pressure on red squirrels to evolve greater biting strength to access the seeds from the cones [42]. In contrast, Douglas squirrels live in the mesic coastal forests west of the Cascade Range, where lodgepole pine is rare or nonexistent and where serotiny is not a seed preservation strategy for any conifer species [39]. Thus, Douglas squirrels feed largely on

seeds from relatively soft-coned conifer species, such as Douglas fir *(Pseudotsuga menziesii),* that do not require biting strengths that are as demanding.

Our study reveals an interesting difference in morphological disparity for both suture complexity and mandible shape between these *Tamiasciurus* species. Red squirrels show greater disparity in both morphospace and suture complexity than Douglas squirrels, indicating their greater range of phenotypic variation in dietary ecomorphology. Morphology can become more variable along an adaptive plateau rather than a sharp peak [60,64]. The greater range of morphological variation in red squirrels than in Douglas squirrels may reflect a wider adaptive plateau and a greater range of viable phenotypes for suture complexity and mandible shape.

Inter-parental ecomorphological differences in sympatry within the hybrid zone

Where red squirrels and Douglas squirrels are sympatric within the hybrid zone, we found ecomorphological divergence patterns that are similar to the patterns observed in allopatry outside the hybrid zone. Indeed, despite competing for the same food resources and experiencing the same dietary biomechanical needs within the hybrid zone, red squirrels maintain greater bite force and suture complexity than Douglas squirrels. The persistence of morphological differences in sympatry suggests divergence in the genetic architecture of some craniomandibular traits are maintained despite potential homogenization effects of backcrossed hybridization and introgression of genetic variation and perhaps some limitations in the role of phenotypic plasticity allowing convergent morphologies in a shared environment. Also of note is the decrease in bite force for both species within the hybrid zone when compared with their conspecifics outside of the hybrid zone. This may suggest weaker

biomechanical demands presented by conifer cones within the hybrid zone. This is potentially due to the hybrid zone containing both large tracts of subalpine forests that are dominated by the soft-coned Engelmann spruce (*Picea engelmannii*), as well as a unique transitional forest that contains a mixture of both mesic and dry conifer species [65]. These findings, however, contrast with our findings on mandibular morphospace, as both species display convergence in mandible shape. We propose that plasticity may have a more critical role in these morphological traits and allows for greater convergence in muscle attachment and mechanical levers in a shared environment between these two squirrel species. Our findings add to previous studies in the *Tamiasciurus* hybrid zone that niche partitioning between red squirrels and Douglas squirrels is either not apparent or very subtle [45,46].

Our findings also do not support expectations of the ecological competitive exclusion theory that species with shared resource requirements cannot stably coexist in an environment [66,67]. Character displacement is one mechanism that allows sympatric species to reduce competition and coexist [8,68-70], as shown in the feeding apparatus of other species in secondary contact [71]. However, our study does not show evidence for character displacement because the ecomorphological divergence in sympatry between the two squirrel species was not greater than the differences between them in allopatry. Interestingly, rather than convergence or character displacement, we found reduced bite force strength in both Douglas squirrels and red squirrels within the hybrid zone compared to their respective allopatric populations. However, the differences in bite force strength between Douglas squirrels and red squirrels remained relatively similar, suggesting that niche partitioning may occur in the hybrid zone, contrary to existing hypotheses for this system [45]. Despite the persistence of

differences between Douglas squirrels and red squirrels within the hybrid zone, we also observe that both species had lower bite force in the sympatric hybrid zone than in allopatry. For red squirrels, this shift may be associated with plasticity, as we would expect that they experience a softer diet in the hybrid zone than the allopatric range; however, we would expect the dietary challenge in the hybrid zone to be greater for Douglas squirrel compared to their allopatric range, yet they demonstrated a decline in bite force. The cause of this shared decline in bite force within the hybrid zone merits exploration in future studies. Another consideration for the lack of character displacement between sympatric red squirrels and Douglas squirrels is the presence of viable hybrids. Hybridization can have a dampening effect on character displacement between co-occurring and competing species where hybrids are viable and possess intermediate phenotypes [47]. However, this is not consistent with the hybrid phenotypes observed here, as hybrids phenotypically mirrored red squirrels rather than being intermediate between the parental species.

Impact of hybridization on ecomorphologies

We find that ecomorphologies of hybrid squirrels follow different general expectations for hybrid phenotypes, including phenotypes that are more like one of the parental species, as well as phenotypes that span across the range of phenotypic variation of both parental species. Incisor strength index, sagittal suture, and premaxillofrontal suture in hybrids are more like co-occurring red squirrels than Douglas squirrels. One explanation for why hybrid morphologies resemble red squirrel morphologies more closely than Douglas squirrel is the genetic dominance of red squirrel alleles underlying these morphological traits. This is consistent with

the lack of correlation between genetic ancestry proportions in hybrids and their incisor strength index or their suture complexity, suggesting further evidence that non-additive genetic variation, such as dominance or environmental effects, contributes to hybrid morphologies. However, a confounding factor that can also produce this pattern is the ecological selection for hybrid morphologies that more closely resemble red squirrel morphologies. Additional studies are needed to distinguish between the effects of genetic effects and selective advantages of red squirrel phenotypes. Addressing this possibility of competition may also shed light on the seemingly stable coexistence of the co-occurring species within the hybrid zone.

In contrast to the incisor strength index and the sagittal and premaxillofrontal sutures, the mandible shapes of hybrids and parental species located within the hybrid zone greatly overlap. One possibility for the similarity in mandible shape between hybrids and both parental species is that plasticity may have a more substantial effect on bony structures of the mandible than tooth shape [72]. Mandibles can be remodeled by their constant interactions with muscles and surrounding tissues, particularly when food ~~harnesses~~ are variable between populations [23,73]. In the *Tamiasciurus* hybrid zone, hybrids and parental species are found in syntopy and gnaw on cones from the same conifer species [39, 46]. Thus, it is conceivable that the shared biomechanical demands on the mandibles of both squirrel species and their hybrids in the hybrid zone, combined with the plasticity of the mandible have led to the overlap in mandibular shape.

Biologists have spent considerable effort in studying both the evolution of craniomandibular ecomorphogical traits, as well as hybrid zones across different mammalian taxa [74-78]. However, very few studies have combined these areas of research in trying to

understand the consequences of admixture on trophic morphologies in mammals. One exception is the well-studied hybrid zone in central Europe between subspecies of house mice (*Mus musculus domesticus and M.m.musculus)* [75]. The phenotypic consequences of hybridization include heterosis of the mandible and skull in hybrids [23,79]. Despite the impressive breadth of research on craniomandibular traits in house mice hybrids, including developmental stability, genetic architecture, and gross morphology [24,80-82]. Thus, ascribing adaptive significance to morphological variation in mice is limited due to the similar ecologies of both mice subspecies. Our study system on *Tamiasciurus* squirrels presents contrasting virtues, and drawbacks to the house mice system as the adaptive significance of morphological variation are more apparently linked to the divergent ecologies between the two squirrel species. However, the larger body size of the squirrels and lower fecundity limit the types of experimental studies that can be conducted to thoroughly understand the developmental and genetic aspects of craniomandibular morphology.

Divergent ecological selection, interspecific competition, and hybridization are all ecological processes that can drive resource partitioning between closely related species and, ultimately, divergence in ecomorphologies. Our findings suggest that divergent selection associated with dietary differences in cone hardness has been an important driver of ecomorphological divergence between *T. douglasii* (soft-cone consumer) and *T. hudsonicus* (hard-cone consumer). Further experimental work, including dietary manipulation, is needed to examine the importance of plasticity. Through our study, we show that significant differences in ecomorphological traits are still observable in closely related taxa within a taxonomic group, like sciurids, that show a high degree of morphological conservatism. Finally, our use of fine-

scale morphological data, such as bite force and suture complexity, provides additional evidence that high-resolution continuous traits are preferable over coarse traits for correctly detecting divergence instead of convergence [83].

Materials and Methods

Specimen Selection

To assess morphological differences between red squirrels, Douglas squirrels, and hybrids, we used a sample of 70 museum specimens across five groups (Table S1): allopatric red squirrels (n=14), hybrid zone red squirrels (n=13), hybrids (n=19), hybrid zone Douglas squirrels (n=10), and allopatric Douglas squirrels (n=14). All specimens in this study are housed at the Burke Museum of Natural History (Table S1). Allopatric specimens were selected such that they were outside the hybrid zone (see Table S1; Fig 1). The selection of specimens from within the hybrid zone was based on previous studies of the *Tamiasciurus* hybrid zone [39, 40]. We also used previously published estimates of admixture proportions for these same specimens [40, 46] based on the program STRUCTURE, an unsupervised clustering method implemented in a Bayesian framework. The estimated individual admixture proportions (Q) represent the fraction of an individual's genome coming from a given population [84] and were used to assign individuals to different genotypic classes (following assignment classes of [85]). We assigned individuals as either pure red squirrel ($Q \geq 0.90$), pure Douglas squirrel ($Q \leq 0.10$), or hybrid ($0.10 < Q < 0.90$).

Bite Force Estimation

Maximal bite force was estimated from complete incisors using an incisor strength index (Z_i) of Z_i = ((antero-posterior length of incisor)^2 x (medio-lateral width of incisor))/6 (as detailed in [27]. This measure is strongly correlated with maximum *in vivo* bite force across Rodentia (($\log_{10}$(bite force) = 0.566 $\log_{10}$ (Z_i) + 1.432, R^2=0.956, [27]), and can be used to estimate maximal bite force for museum specimens where *in vivo* bite force data are unavailable. The anteroposterior length and the medio-lateral width of the lower right incisor of each specimen was measured using a Dino-Lite Edge AM7915 digital microscope by a single investigator (DMP) (Fig 7A). Because bite force scales allometrically with body size [27, 86, 87], bite force was converted to a bite force quotient (BFQ) using the residuals of the regression between $\log_{10}$(bite force) and $\log_{10}$(body mass) for all squirrel specimens in this study (following the method of [87]. Bite force data were analyzed with an ANCOVA (using sex as a covariate) in RStudio version 1.4.1103 [88]. When ANCOVA results were significant, post-hoc pairwise comparisons were performed to test for differences among squirrel groups. To test for the potential effects of hybrid admixture on bite force estimation, we used a linear least squares regression of bite force and admixture score. We predicted that (1) red squirrels have a stronger bite force than Douglas squirrels when compared both allopatrically and sympatrically, and (2) hybrids have a phenotype intermediate between the sympatric parental species.

Fig 7. (A). Measurements of incisors in lateral and occlusal views and equation of the incisor strength index (Zi) used as a proxy for bite force. **(B).** Schematic of cranial sutures used in this study with the nasofrontal suture (NF) in red, the premaxillofrontal suture (PMF) in brown, the maxillofrontal suture (MF) in yellow, the coronal suture (C) in pink, and the sagittal suture (S) in

green. **(C).** Position of landmarks (large red circles) and semilandmarks (small black circles) on the mandible in lateral view. Descriptions of landmarks and semilandmarks are provided in Tables S2-3.

Suture Morphology

We quantified a complementary measure of bite force and skull suture complexity for all museum specimens with uninterrupted sutures of intact skulls. We measured five suture lines (coronal, sagittal, nasofrontal, maxillofrontal, and premaxillofrontal sutures; Fig 7B) for each specimen. To do this, we photographed the dorsal view of the cranium from a fixed distance using a Canon EOS Rebel T6i. We then made vector graphics of sutures in Inkscape v0.93 (inkscape.org) by tracing the total length of the path of each suture on the images. We chose the right side of the crania to measure the maxillofrontal and premaxillofrontal sutures unless these were damaged, in which case, we measured the sutures on the left side. The complexity of each suture was quantified as a length ratio (LR), whereby the total length of the suture path is divided by the linear distance between the start and end [32,89,90]. Suture complexity data were analyzed with an ANCOVA, with sex as a covariate. When ANCOVA results were significant, post-hoc pairwise comparisons were performed to test for differences among squirrel groups. To test for a possible multivariate effect of suture complexity, all five sutures were also analyzed together using a principal component analysis (PCA). We used ANOVAs to test differences in PC1 and PC2 scores among squirrel groups. Finally, to test for the potential effects of hybrid admixture on suture complexity, we performed linear least squares regressions of suture complexity and admixture score for all five sutures. We predicted that (1)

red squirrels have more complex sutures than Douglas squirrels when compared both allopatrically and sympatrically, and (2) hybrids have a phenotype intermediate between the sympatric parental species.

Geometric Morphometrics

To test for morphological divergence and morphospace overlap among the squirrel groups studied, we performed a geometric morphometric analysis of lower jaw shape including all complete specimens. The dentaries were photographed in lateral view on the same plane at a fixed distance using a Canon EOS Rebel T6i camera. For each specimen, 15 landmarks and 45 semilandmarks were digitized in TpsDig2 software [91]. The landmarks scheme represents homologous locations ~~corresponding to dietarily relevant features~~, adapted from schemes used for squirrels [35] and hybridizing mice [24]. The semilandmarks used are equidistant points resampled to the lowest number, properly encapsulating the shape of the curve from homologous curves (Fig 7C, Landmark Description in Table S2 and Table S3). Landmark data were imported and analyzed using the R package geomorph v. 4.0.1 [92]. Semilandmarks were registered as sliders and slid according to the bending energy criterion [93]. Landmarks and semilandmarks were subjected to Generalized Procrustes Analysis (GPA) [94,95]. A PCA was performed on the GPA data to explore group differences in shape data. Only significant axes were retained for subsequent analyses, as determined by Monte Carlo randomization test run in biostats [96]. Procrustes ANOVAs were performed on these data in geomorph to assess the relationships between shape, size, sex, and group, with size represented by the log10-transformed centroid sizes from GPA. Pairwise comparisons were generated from these data in

geomorph. We predicted that red squirrels would occupy a distinct morphospace from Douglas squirrels, with hybrids occupying an intermediate, and potentially overlapping, morphospace between the parental species.

Morphospace overlap between groups was analyzed using nicheROVER [97] using PC1 and PC2 scores, which represent the shape, and log10-transformed centroid size, which represents size. This analysis provides the probabilities of overlap between squirrel group pairs by taking advantage of a method developed to analyze multidimensional niche space overlap [98,99]. Distributions were generated from the posterior distributions of 3,000 permutations of overlap probability. We predicted that for each species, the allopatric squirrels would have a high probability of overlap with hybrid zone conspecifics but low overlap with heterospecific populations, whereas hybrids would have a moderate to high probability of overlap with the other four squirrel groups.

Acknowledgements

We thank Jeff Bradley (University of Washington) for help and access to specimens in the University of Washington Burke Museum collections. We additionally thank Alec Sheets (The Ohio State University) for feedback and discussion on earlier versions of this manuscript.

References

1. Jones K, Law CJ. Differentiation of craniomandibular morphology in two sympatric Peromyscus mice (Cricetidae: Rodentia). Mammal Res. 2018; 63: 277–283. doi: 10.1007/s13364-018-0364-2.
2. Feller AF, Selz OM, McGee MD, Meier JI, Mwaiko S, Seehausen O. Rapid generation of ecologically relevant behavioral novelty in experimental cichlid hybrids. Ecol Evol. 2020; 10: 7445–7462. doi: 10.1002/ece3.6471.
3. Selz OM, Seehausen O. Interspecific hybridization can generate functional novelty in cichlid fish. Proc Royal Soc B. 2019; 286: 20191621. doi: 10.1098/rspb.2019.1621.
4. Nürnberger B, Barton N, MacCallum C, Gilchrist J, Appleby M. Natural selection on quantitative traits in the *Bombina* hybrid zone. Evolution. 1995; 49: 1224–1238. doi: 10.2307/2410447.
5. Milano ER, Kenney AM, Juenger TE. Adaptive differentiation in floral traits in the presence of high gene flow in scarlet gilia (*Ipomopsis aggregata*). Mol Ecol. 2016; 25: 5862–5875. doi: 10.1111/mec.13752.
6. Knief U, Bossu CM, Saino N, Hansson B, Poelstra J, Vijay N, et al. Epistatic mutations under divergent selection govern phenotypic variation in the crow hybrid zone. Nat Ecol Evol. 2019; 3: 570–576. doi: 10.1038/s41559-019-0847-9.
7. Pfennig KS, Pfennig DW. Character displacement: ecological and reproductive responses to a common evolutionary problem. Q Rev Biol. 2009; 84: 253–276. doi: 10.1086/605079.
8. Pfennig DW, Pfennig PF. Character displacement and the origins of diversity. Am Nat. 2010; 176: S26–S44. doi: 10.1086/657056.
9. Reifová R Reif J, Antczak M, Nachman MW. Ecological character displacement in the face of geneflow: evidence from two species of nightingales. BMC Evol Biol. 2011; 11: 138. doi: 10.1186/1471-2148-11-138.
10. Cody ML. Convergent characteristics in sympatric species - a possible relation to interspecific competition and aggression. Condor. 1969; 71: 223–239. doi: 10.2307/1366300.
11. Cody ML. Character convergence. Annu Rev Ecol Evol Syst. 1973; 4: 189–211. doi: 10.1146/annurev.es.04.110173.001201.
12. Leary CJ. Evidence of convergent character displacement in release vocalizations of *Bufo fowleri* and *Bufo terrestris* (Anura; Bufonidae). Anim Behav. 2001; 61: 431–438. doi: 10.1006/anbe.2000.1597.
13. Parsons KJ, Son YH, Albertson CR. Hybridization promotes evolvability in African cichlids: Connections between transgressive segregation and phenotypic integration. Evol Biol. 2011; 38: 306–315. doi: 10.1007/s11692-011-9126-7.
14. Selz OM, Lucek K, Young KA, Seehausen O. Relaxed trait covariance in interspecific cichlid hybrids predicts morphological diversity in adaptive radiations. J Evol Biol. 2014; 27: 11–24. doi: 10.1111/jeb.12283.

15. Wilde GR, Echelle AA. Morphological variation in intergrade pupfish populations from the Pecos River, Texas, U.S.A. J Fish Biol. 1997; 50: 523–539. doi: 10.1111/j.1095-8649.1997.tb01947.x.
16. Loy A, Capula M, Palombi A, Capanna E. Genetic and morphometric evidence of introgression between two species of moles (Insectivora: *Talpa europaea* and *Talpa romana*) in central Italy. J Zool. 2001; 254: 229–238. doi: 10.1017/S0952836901000747.
17. Klingenberg CP, Leamy LJ, Routman EJ, Cheverud JM. Genetic architecture of mandible shape in mice: effects of quantitative trait loci analyzed by geometric morphometrics. Genetics. 2001; 157: 785–802. doi: 10.1093/genetics/157.2.785.
18. Klingenberg CP, Leamy LJ, Cheverud JM. Integration and modularity of quantitative trait locus effects on geometric shape in the mouse mandible. Genetics. 2004; 166: 1909–1921. doi: 10.1093/genetics/166.4.1909.
19. Workman MS, Leamy LJ, Routman EJ, Cheverud JM. Analysis of quantitative trait locus effects on the size and shape of mandibular molars in mice. Genetics. 2002; 160: 1573–1586. doi: 10.1093/genetics/161.3.1355.
20. Monti L, Baylac M, Lalanne-Cassou B. Elliptic Fourier analysis of the form of genitalia in two *Spodoptera* species and their hybrids (Lepidoptera: Noctuidae). Biol J Linn Soc. 2001; 72: 391–400. doi: 10.1111/j.1095-8312.2001.tb01325.x.
21. Valentin A, Sévigny J-M, Chanut J-P. Geometric morphometrics reveals body shape differences between sympatric redfish *Sebastes mentella*, *Sebastes fasciatus* and their hybrids in the Gulf of St Lawrence. J Fish Biol. 2002; 60: 857–875. doi: 10.1111/j.1095-8649.2002.tb02414.x.
22. Nolte AW, Sheets HD. Shape based assignments tests suggest transgressive phenotypes in natural sculpin hybrids (Teleostei, Scorpaeniformes, Cottidae). Front Zool. 2005; 2: 11. doi: 10.1186/1742-9994-2-11.
23. Renaud S, Alibert P, Auffray J-C. Mandible shape in hybrid mice. Naturwissenschaften. 2009; 96: 1043–1050. doi: 10.1007/s00114-009-0563-4.
24. Renaud S, Alibert P, Auffray J-C. Modularity as a source of new morphological variation in the mandible of hybrid mice. BMC Evol Biol. 2012; 12, 141. doi: 10.1186/1471-2148-12-141.
25. Konuma J, Sota T, Chiba S. A maladaptive intermediate form: a strong trade-off revealed by hybrids between two forms of a snail feeding beetle. Ecology. 2013; 94: 2638–2644. doi: 10.1890/12-2041.1.
26. Badyaev AV. The beak of the other finch: Coevolution of genetic covariance structure and developmental modularity during adaptive evolution. Philos Trans R Soc. 2010; 365: 1111–1126. doi: 10.1098/rstb.2009.0285.
27. Freeman PW, Lemen CA. A simple morphological predictor of bite force in rodents. J Zool. 2008; 275: 418-422. doi: 10.1111/j.1469-7998.2008.00459.x.
28. Herring SW, Mucci RJ. In vivo strain in cranial sutures: The zygomatic arch. J Morphol. 1991; 207: 225–239. doi: 10.1002/jmor.1052070302.

29. Herring SW, Teng S. Strain in the braincase and its sutures during function. Am J Phys Anthropol. 2000; 112: 575–593. doi: 10.1002/1096-8644(200008)112:4<575::AID-AJPA10>3.0.CO;2-0.
30. Sun Z, Lee E, Herring SW. Cranial sutures and bones: Growth and fusion in relation to masticatory strain. *Anat Rec. 2004; 276(2*): 150–161. doi: 10.1002/ar.a.20002.
31. Moazen M, Curtis N, O'Higgins P, Jones MEH, Evans SE, Fagan MJ. Assessment of the role of sutures in a lizard skull: a computer modeling study. Proc Royal Soc B. 2008; 276: 39–46. doi: 10.1098/rspb.2008.0863.
32. Buezas G, Becerra F, Vassallo A. Cranial suture complexity in caviomorph rodents (Rodentia; Ctenohystrica). J Morphol. 2017; 278: 1125-1136. doi: 10.1002/jmor.20699.
33. Rager L, Hautier L, Forasiepi A, Goswami A, Sanchez-Villagra M. Timing of cranial suture closure in placental mammals: phylogenetic patterns, intraspecific variation, and comparison with marsupials. J Morphol. 2014; 275: 125–140. doi: 10.1002/jmor.20203.
34. White HE, Goswami A, Tucker AS. The intertwined evolution and development of sutures and cranial morphology. Front Cell Dev Biol. 2021; *9*: 690. doi: 10.3389/fcell.2021.653579.
35. Casanovas-Vilar I, van Dam J. Conservatism and adaptability during squirrel radiation: What is mandible shape telling us? PLoS ONE, 2013; 8:e61298. doi: 10.1371/journal.pone.0061298.
36. Becerra F, Echeverría AI, Casinos A, Vassallo AI. Another bites the dust: Bite force and ecology in three caviomorph rodents (Rodentia, Hystricognathi). J Exp Zool A Ecol Genet Physiol. 2014; 321: 220–232. doi: 10.1002/jez.1853.
37. Tanner JB, Zelditch ML, Lundrigan BL, Holekamp KE. Ontogenetic change in skull morphology and mechanical advantage in the spotted hyena (*Crocuta crocuta*). J Morphol. 2010; 271: 353–365. doi: 10.1002/jmor.10802.
38. Zelditch ML, Ye J, Mitchell JS, Swiderski DL. Rare ecomorphological convergence on a complex adaptive landscape: body size and diet mediate evolution of jaw shape in squirrels (Sciuridae). Evolution. 2017; 71, 633–649. doi: 10.1111/evo.13168.
39. Smith CC. The adaptive nature of social organization in the genus of three squirrels *Tamiasciurus*. Ecol Monogr. 1968; 38: 31-63. doi: 10.2307/1948536.
40. Chavez AS, Saltzberg CJ, Kenagy GJ. Genetic and phenotypic variation across a hybrid zone between ecologically divergent tree squirrels (*Tamiasciurus*). *Mol Ecol. 2011; 20*: 3350-3366. doi: 10.1111/j.1365-294X.2011.05184.x.
41. Chavez AS, Maher SP, Arbogast BS, Kenagy GJ. Diversification and gene flow in nascent lineages of island and mainland North American tree squirrels (*Tamiasciurus*). Evolution. 2014; 68: 1094–1109. doi: 10.1111/evo.12336.
42. Smith CC. The coevolution of pine squirrels (*Tamiasciurus*) and conifers. Ecol Monogr. 1970; 40: 349-371. doi: 10.2307/1942287.
43. Talluto MV, Benkman CW. Conflicting selection from fire and seed predation drives fine-scaled phenotypic variation in a widespread North American conifer. Proc Natl Acad Sci U.S.A., 2014; 111: 9543–9548. doi: 10.1073/pnas.1400944111.

44. Benkman CW, Siepielski AM. A keystone selective agent? Pine squirrels and the frequency of serotiny in lodgepole pine. Ecology. 2004; 85: 2082–2087. doi: 10.1890/04-0177.
45. Smith CC. The indivisible niche of *Tamiasciurus*: an example of nonpartitioning of resources. *Ecol Monogr. 1981; 51*: 343–363. doi: 10.2307/2937278.
46. Fotis AT, Patel S, Chavez AS. Habitat-based isolating barriers are not strong in the speciation of ecologically divergent squirrels (*Tamiasciurus douglasii* and *T. hudsonicus*). Behav Ecol Sociobiol. 2020; 74(3): 1-14. doi: 10.1007/s00265-020-2814-5.
47. Aguilée R, de Becdelièvre B, Lambert A, Claessen D. Under which conditions is character displacement a likely outcome of secondary contact? *J Biol Dyn. 2011; 5:* 135–146. doi: 10.1080/17513758.2010.491559.
48. Anderson RA, Macbrayer LD, Herrel A. Bite force in vertebrates: opportunities and caveats for use of a non-pareil whole-animal performance measure. Biol J Linn Soc. 2008; 93: 709-720. doi: 10.1111/j.1095-8312.2007.00905.x.
49. Aguirre LF, Herrel A, van Damme R, Matthysen E. Ecomorphological analysis of trophic niche partitioning in a tropical savanna bat community. Proc Royal Soc B. 2002; 269: 1271–1278. doi: 10.1098/rspb.2002.2011.
50. Aguirre LF, Herrel A, van Damme R, Matthysen E. The implications of food hardness for diet in bats. Funct Ecol. 2003; 17: 201–212. doi: 10.1046/j.1365-2435.2003.00721.x.
51. Herrel A, Podos K, Huber SK, Hendry AP. Bite performance and morphology in a population of Darwin's finches: implications for the evolution of beak shape. Funct Ecol. 2005; 19: 43–48. doi: 10.1111/j.0269-8463.2005.00923.x.
52. Santana SE, Dumont ER, Davis JL. Mechanics of bite force production and its relationship to diet in bats. Funct. Ecol. 2010; 24: 776–784. doi: 10.1111/j.1365-2435.2010.01703.x.
53. Cornette R, Tresset A, Houssin C, Pascal M, Herrel A. Does bite force provide a competitive advantage in shrews? The case of the greater white-toothed shrew. Biol J Linn Soc. 2015; 114: 795–807. doi: 10.1111/bij.12423.
54. Herrel A, Van Damme R, Vanhooydonck B, De Vree E. The implications of bite performance for diet in two species of lacertid lizards. Can J Zool. 2001; 79: 662-670. doi: 10.1139/z01-031.
55. Nogueira MR, Peracchi AL, Monteiro LR. Morphological correlates of bite force and diet in the skull and mandible of phyllostomid bats. *Funct Ecol. 2009; 23*: 715–723. doi: 10.1111/j.1365-2435.2009.01549.x.
56. Byron C, Segreti M, Hawkinson K, Herman K, Patel S. Dietary material properties shape cranial suture morphology in the mouse calvarium. J Anat. 2018; 233: 807-813. doi: 10.1111/joa.12888.
57. Hautier L, Fabre PH, Michaux J. Mandible shape and dwarfism in squirrels (Mammalia, Rodentia): interaction of allometry and adaptation. Naturwissenschaften, 2009; 96: 725–730. doi: 10.1007/s00114-009-0519-8.
58. Swiderski DL, Zelditch ML. (2010). Morphological diversity despite isometric scaling of lever arms. Evol Biol. 2010; 37: 1–18. doi: 10.1007/s11692-010-9081-8.

59. Zelditch ML, Li J, Tran LAP, Swiderski DL. Relationships of diversity, disparity, and their evolutionary rates in squirrels (Sciuridae). Evolution, 2015; 69: 1284–1300. doi: 10.1111/evo.12642.
60. Zelditch ML, Li J, Swiderski DL. Stasis of functionally versatile specialists. Evolution, 2020; 74: 1356–1377. doi: 10.1111/evo.13956.
61. Muir PS, Lotan JE. Disturbance history and serotiny of *pinus contorta* in western Montana. Ecology. 1985; 66: 1658–1668. doi: 10.2307/1938028.
62. Benkman CW, Smith JW, Maier M, Hansen L, Talluto MV. Consistency and variation in phenotypic selection exerted by a community of seed predators. Evolution. 2013; 67: 157–169. doi: 10.1111/j.1558-5646.2012.01736.x.
63. Parker AL, Benkman CW. Enhanced seed defenses potentially relax selection by seed predators against serotiny in lodgepole pine. Ecol Evol. 2020; 10: 6001–6008. doi: 10.1002/ece3.6339.
64. Maestri R, Monteiro LR, Fornel R, Upham NS, Patterson BD, de Freitas TR. The ecology of a continental evolutionary radiation: is the radiation of sigmodontine rodents adaptive? Evolution, 2017; 71: 610–632. doi: 10.1111/evo.13155.
65. Franklin JF, Dyrness CT. Natural vegetation of Oregon and Washington. Corvallis, OR: Oregon State University Press. 1988.
66. Gause GF. Experimental analysis of Vito Volterra's mathematical theory of the struggle for existence. Science, 1934; 79: 16–17. doi: 10.1126/science.79.2036.16.b.
67. Hardin G.The competitive exclusion principle. Science, 1960; 131: 1292–1297. doi: 10.1126/science.131.3409.1292.
68. Brown WL, Wilson EO. Character displacement. *Syst Zool. 1956; 5*: 49–64. doi: 10.2307/2411924.
69. Grant PR. Convergent and divergent character displacement. Biol J Linn Soc. 1972; 4: 39-68. doi: 10.1111/j.1095-8312.1972.tb00690.x.
70. Schluter D. The ecology of adaptive radiation*.* Oxford University Press. Oxford, U. K. 2000.
71. Grant PR, Grant BR. Evolution of character displacement in Darwin's finches. Science. 2006; 313: 224–226. doi: 10.1126/science.1128374.
72. Renaud S, Auffray J-C. (2013). The direction of main phenotypic variance as a channel to evolution: cases in murine rodents. Hystrix. 2013; 24: 85–93. doi: 10.4404/hystrix-24.1-6296.
73. Mavropoulos A, Bresin A, Kiliaridis S. Morphometric analysis of the mandible in growing rats with different masticatory functional demands: adaptation to an upper posterior bite block. Eur J Oral Sci. 2004; 112: 259–266. doi: 10.1111/j.1600-0722.2004.00129.x.
74. Shurtliff QR. Mammalian hybrid zones: a review. Mamm Rev. 2013; 43: 1–21. doi: 10.1111/j.1365-2907.2011.00205.x.
75. Warren KA, Ritzman TB, Humphreys RA, Percival CJ., Hallgrímsson B, Ackermann RR. Craniomandibular form and body size variation of first generation mouse hybrids: a

model for hominin hybridization. J Hum Evol. 2018; 116: 57-74. doi: 10.1016/j.jhevol.2017.12.002.
76. Tamagnini D, Meloro C, Raia P, Maiorano L. Testing the occurrence of convergence in the cranio-mandibular shape evolution of living carnivorans. Evolution. 2021; 75: 1738–1752. doi: 10.1111/evo.14288.
77. Cassini GH, Toledo N. An ecomorphological approach to craniomandibular integration in neotropical deer. J Mamm Evol. 2020; 28: 111–123. doi: 10.1007/s10914-020-09499-5.
78. Tse YT, Calede JJM. (2021). Quantifying the link between craniodental morphology and diet in the Soricidae using geometric morphometrics. Biol J Linn Soc. 2021; 133: 28-46. doi: 10.1093/biolinnean/blab005.
79. Debat V, Alibert P, David P, Paradis E, Auffray J-C. Independence between developmental stability and canalization in the skull of the house mouse. Proc Royal Soc B. 2002; 267: 423–430. doi: 10.1098/rspb.2000.1017.
80. Leamy L. Morphometric studies in inbred and hybrid house mice. V. Directional and fluctuating asymmetry. Am Nat. 1984; 123: 579–593. doi: 10.1086/284225.
81. Alibert P, Renaud S, Dod B, Bonhomme F, Auffray J-C. Fluctuating asymmetry in the *Mus musculus* hybrid zone: a heterotic effect in disrupted co-adapted genomes. Proc Royal Soc B. 1994; 258: 53–59. doi: 10.1098/rspb.1994.0141.
82. Pallares LF, Turner LM, Tautz D. Craniofacial shape transition across the house mouse hybrid zone: Implications for the genetic architecture and evolution of between-species differences. Dev Genes Evol. 2016; 226: 173–186. doi: 10.1007/s00427-016-0550-7.
83. Kohli BA, Jarzyna MA. Pitfalls of ignoring trait resolution when drawing conclusions about ecological processes. Glob Ecol Biogeogr. 2021; 30: 1139–1152. doi: 10.1111/geb.13275.
84. Pritchard JK, Stephens M, Donnelly P. Inference of population structure using multilocus genotype data. Genetics. 2000; 155: 945–959. doi: 10.1093/genetics/155.2.945.
85. Vähä JP, Primmer CR. Efficiency of model-based Bayesian methods for detecting hybrid individuals under different hybridization scenarios and with different numbers of loci. Mol Ecol. 2006; 15: 63–72. doi: 10.1111/j.1365-294X.2005.02773.x.
86. Meers MB. Maximum bite force and prey size of *Tyrannosaurus rex* and their relationships to the inference of feeding behavior. Hist Biol. 2002; 16: 1-12. doi: 10.1080/0891296021000050755.
87. Wroe S, McHenry C, Thomason J. Bite club: Comparative bite force in big biting mammals and the prediction of predatory behaviour in fossil taxa. Proc Royal Soc B. 2005; 272 (1563): 619–625. doi: 10.1098/rspb.2004.2986.
88. RStudio Team. RStudio: Integrated Development for R. Boston, MA: RStudio Inc. 2021.
89. Jaslow CR. Sexual dimorphism of cranial suture complexity in wild sheep (*Ovis orientalis*). Biol J Linn Soc. 1989; 95(4): 273–284. doi: 10.1111/j.1096-3642.1989.tb02312.x.

90. White HE, Clavel J, Tucker AS, Goswami A. A comparison of metrics for quantifying cranial suture complexity. J R Soc Interface. 2020; 17: 20200476. doi: 10.1098/rsif.2020.0476.
91. Rohlf FJ. tpsDig, digitize landmarks and outlines, version 2.32. Department of Ecology and Evolution, State University of New York at Stony Brook. 2005.
92. Adams DC, Otárola-Castillo E. (2013). Geomorph: An R package for the collection and analysis of geometric morphometric shape data. Methods Ecol Evol. 2013; 4: 393–399. doi: 10.1111/2041-210X.12035.
93. Perez SI, Bernal V, Gonzalez PN. Differences between sliding semi-landmark methods in geometric morphometrics, with an application to human craniofacial and dental variation. J Anat. 2006; 208: 769-784. doi: 10.1111/j.1469-7580.2006.00576.x.
94. Bookstein FL. Morphometric tools for landmark data: geometry and biology. Cambridge UK: Cambridge University Press. 1997. doi: 10.1017/CBO9780511573064.
95. Zelditch ML, Swiderski DL, Sheets HD. Geometric morphometrics for biologists: a primer. New York: Academic Press. 2012. doi: 10.1016/B978-0-12-778460-1.X5000-5.
96. McGarigal K. Biostats. 2015.
97. Swanson HK, Lysy M, Power M, Stasko AD, Johnson JD, Reist JD. A new probabilistic method for quantifying n-dimensional ecological niches and niche overlap. Ecology. 2015; 96: 318-324. doi: 10.1890/14-0235.1.
98. Hedrick BP. Inter- and intraspecific variation in *Artibeus* species complex demonstrates size and shape partitioning among species. Peer J. 2021; 9:e11777. doi: 10.7717/peerj.11777.
99. Machado FA, Zahn TMG, Marroig G. Evolution of morphological integration in the skull of Carnivora (Mammalia): Changes in Canidae lead to increased evolutionary potential of facial traits. Evolution. 2018; 72: 1399-1419. doi: 10.1111/evo.13495.

Figure 1 Click here to access/download;Figure;Fig1.jpg

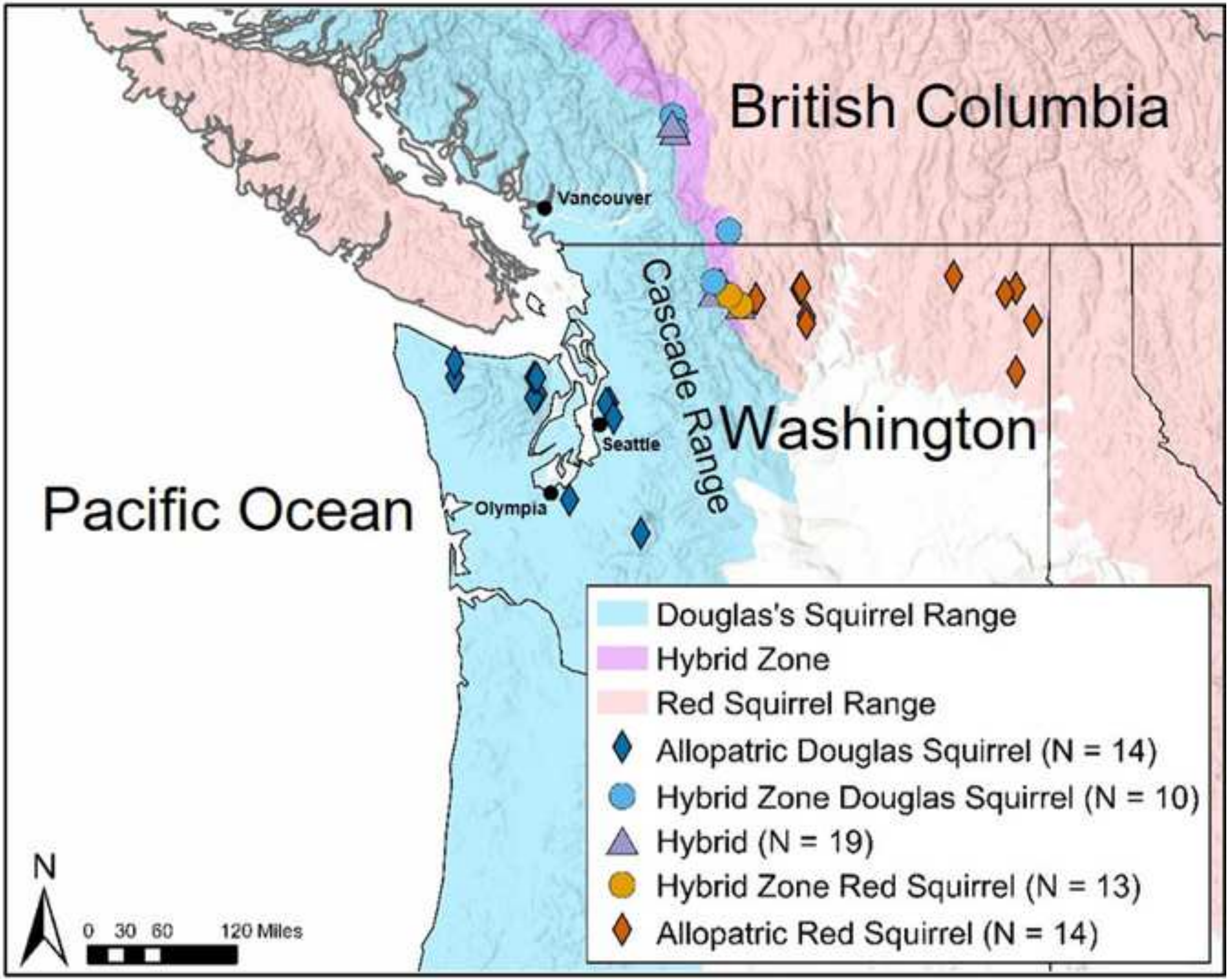

Figure 2

Click here to access/download;Figure;Fig2.jpg

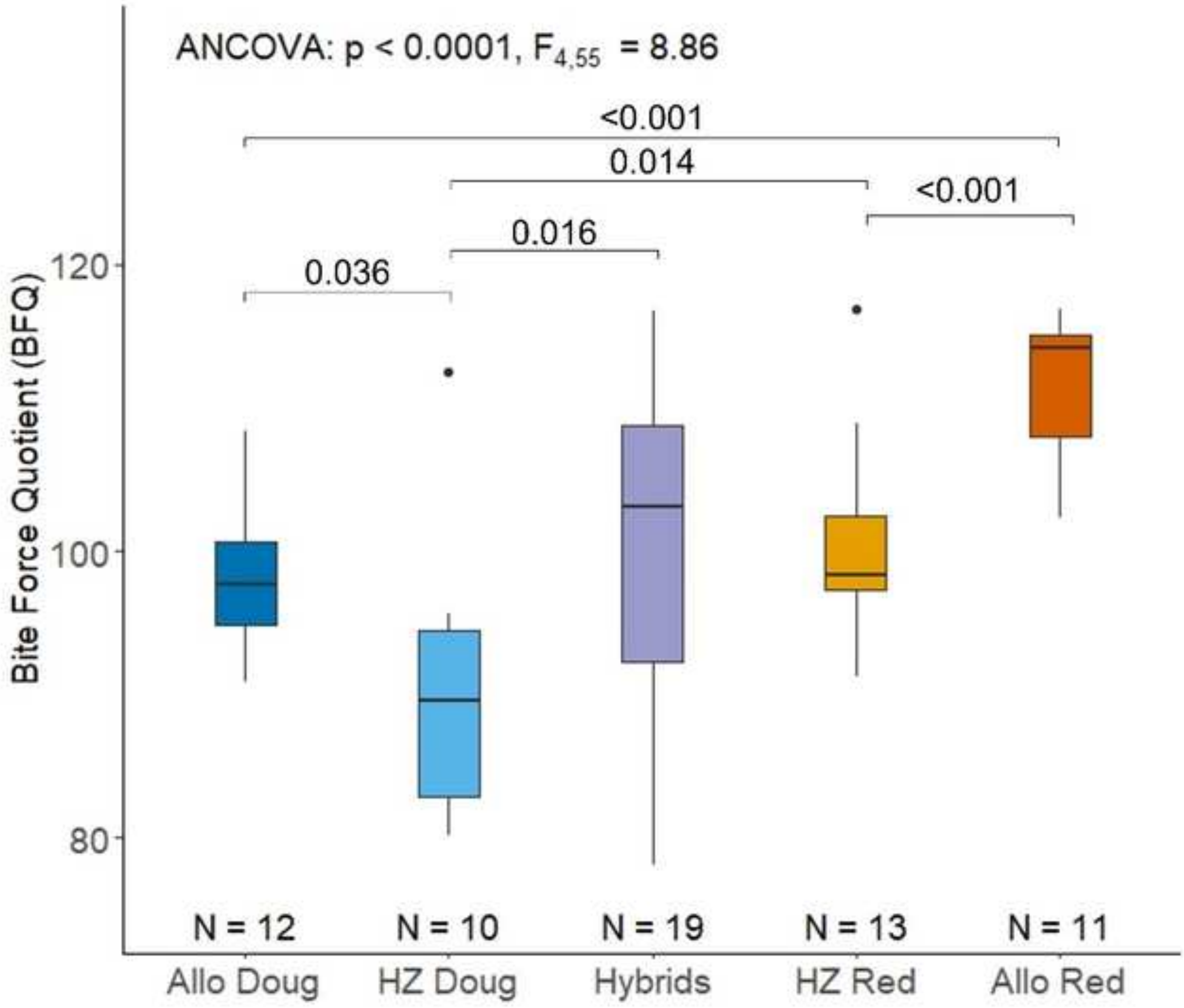

Figure 3

Click here to access/download;Figure;Fig3.jpg

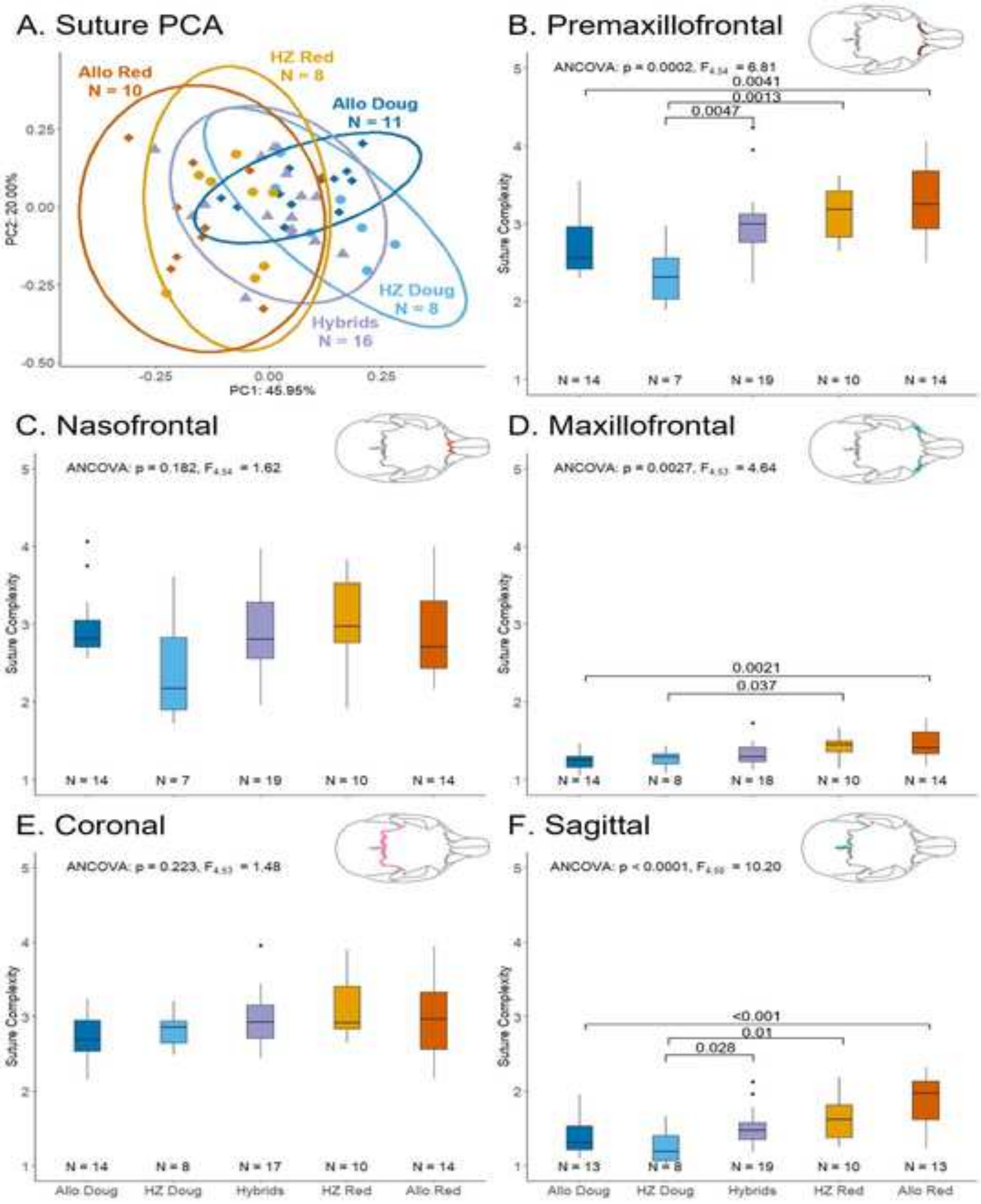

Figure 4

Click here to access/download;Figure;Fig4.jpg

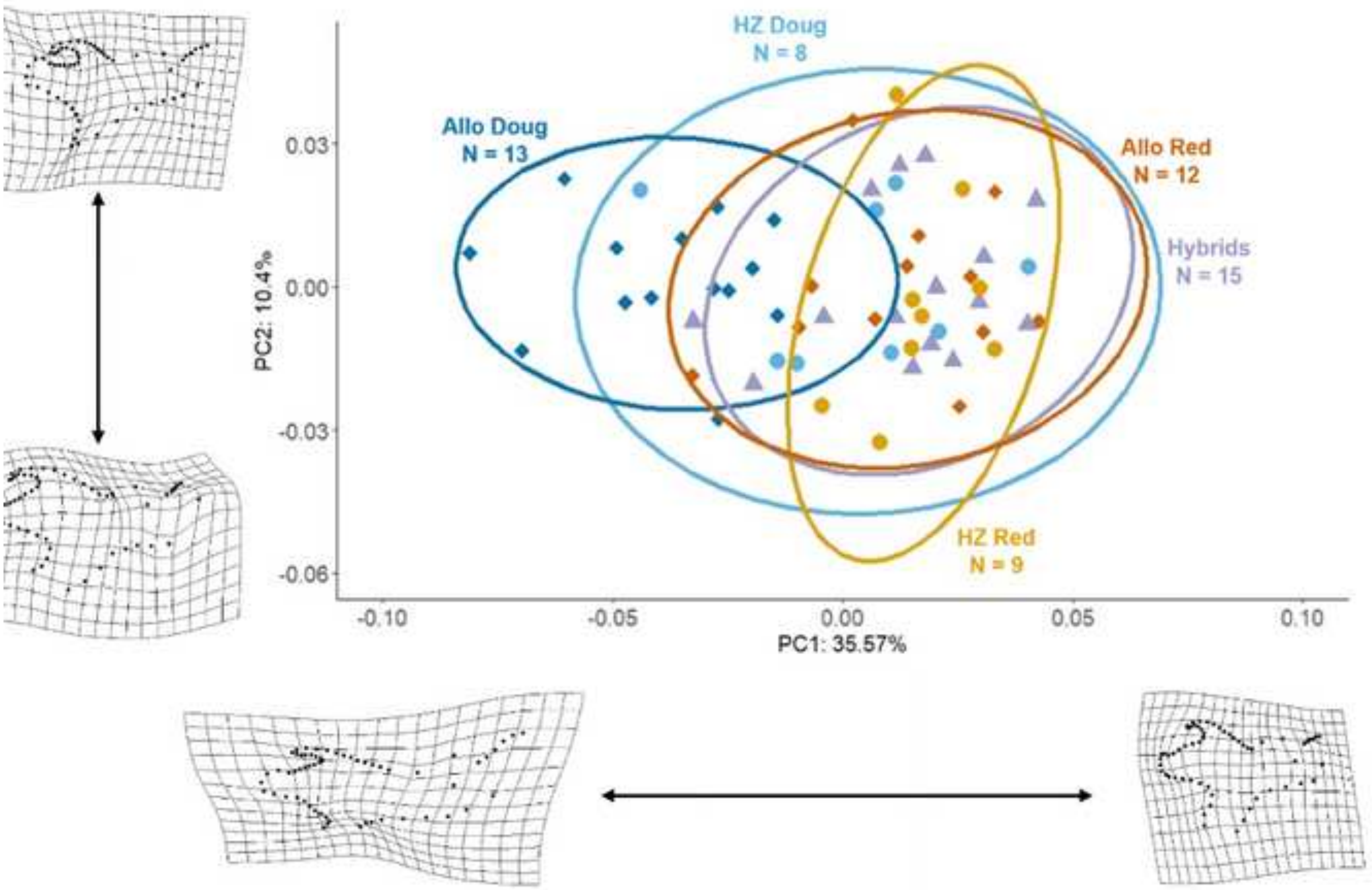

Figure 5

Click here to access/download;Figure;Fig5.jpg

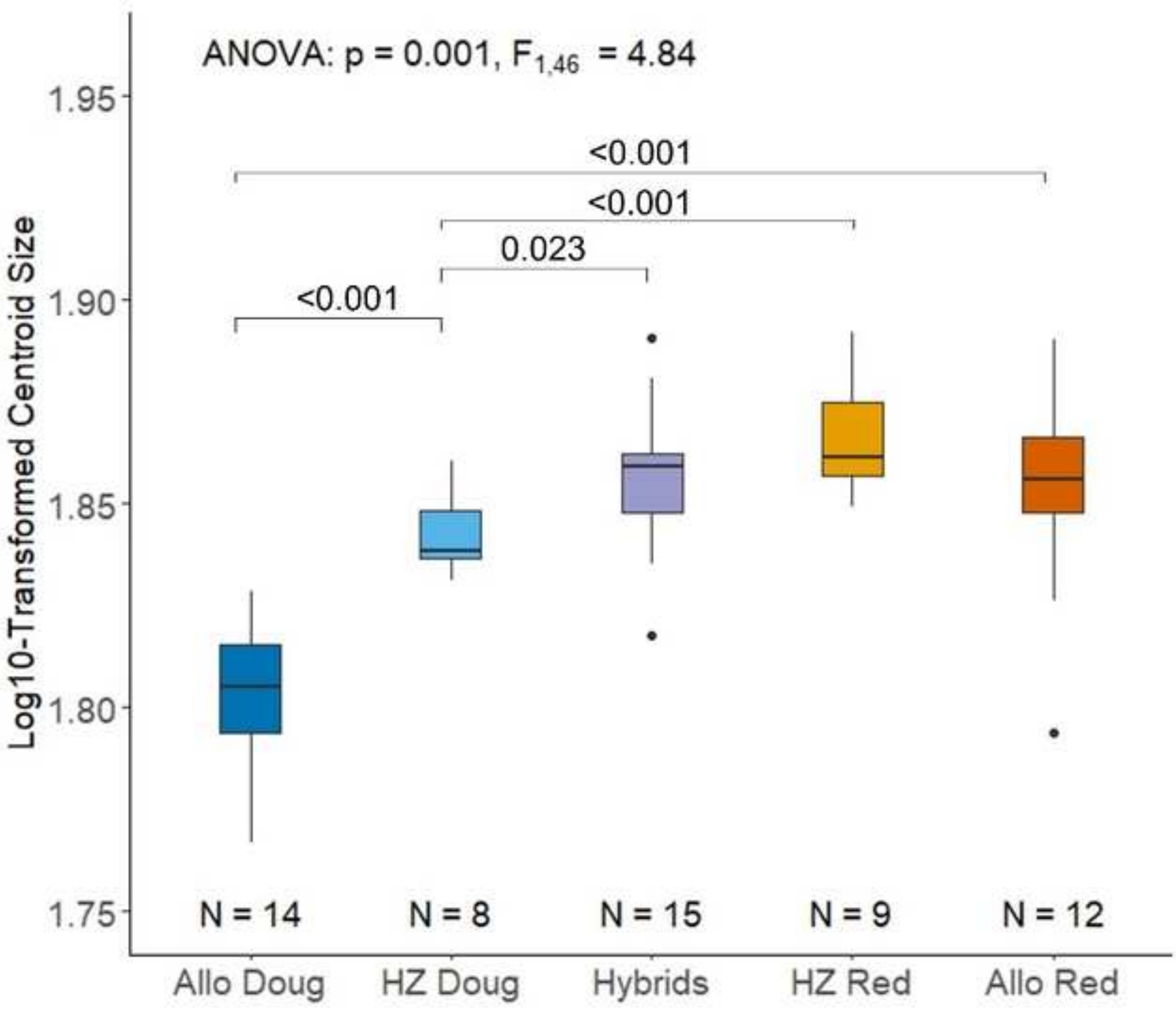

Figure 6

Click here to access/download;Figure;Fig6.jpg

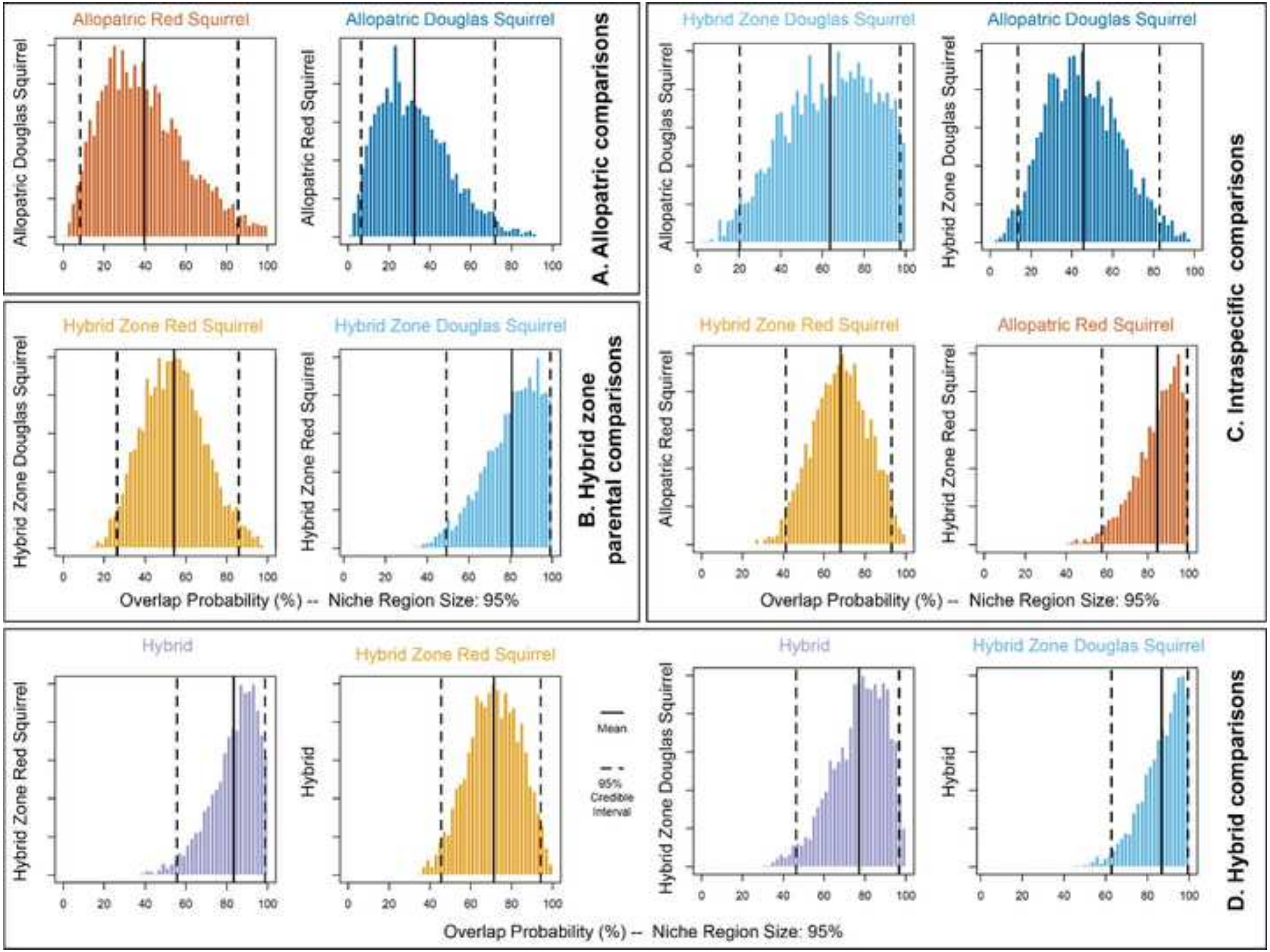

Figure 7

Click here to access/download;Figure;Fig7.jpg

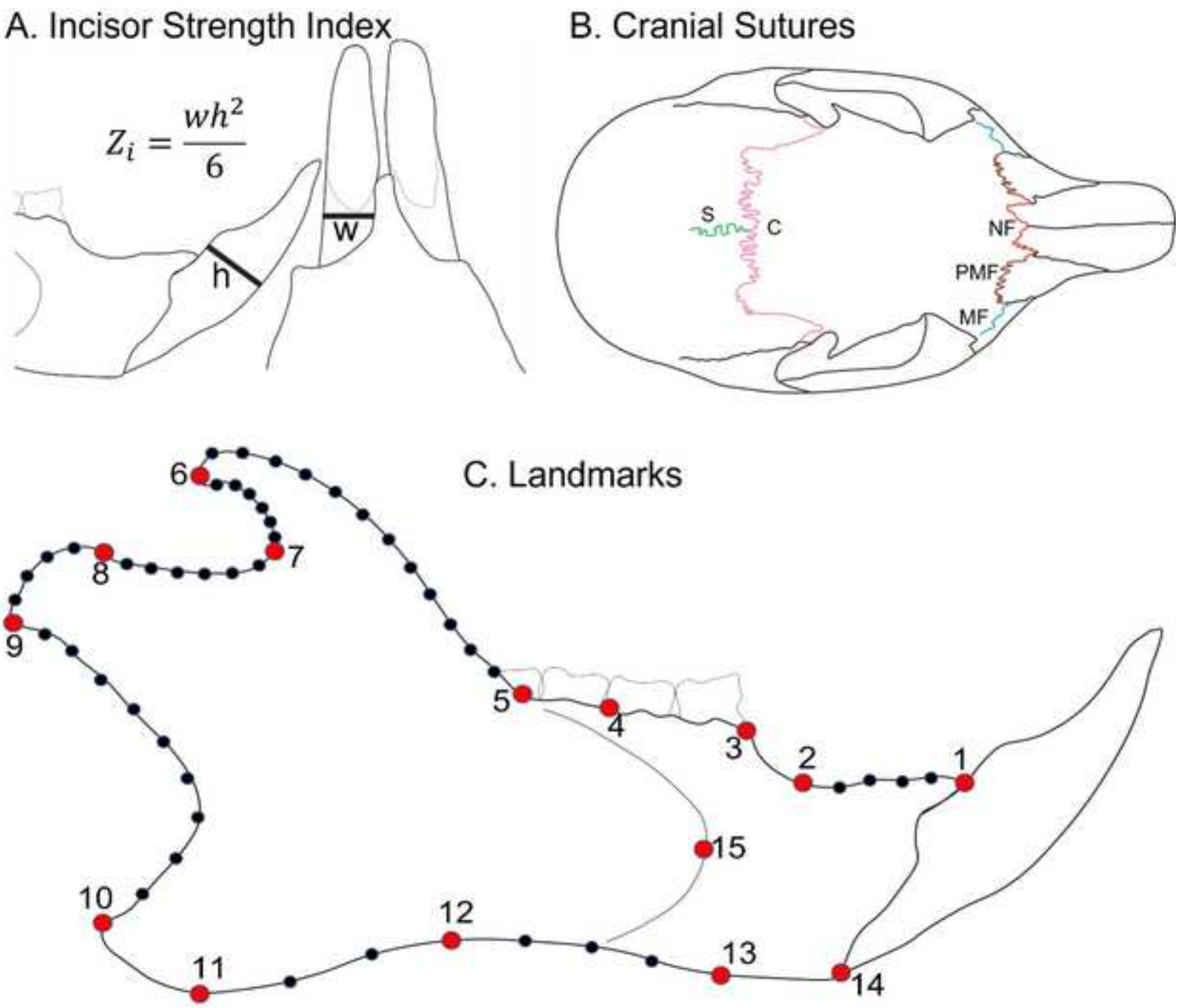

Table S1

Click here to access/download
Supporting Information
Poorboy_etal_Table S1.xlsx

Table S2

Click here to access/download
Supporting Information
TableS2.docx

Table S3

Click here to access/download
Supporting Information
TableS3.docx

Figure S1

Click here to access/download
Supporting Information
FigS1.docx
